# Intracranial Hemorrhage After Reduction Malarplasty: A Narrative Review Focusing on Surgical Technique

**DOI:** 10.3390/jcm15145609

**Published:** 2026-07-17

**Authors:** Myoung Soo Kim

**Affiliations:** Division of Neurosurgery, Seoul Regional Trauma Center, National Medical Center, Seoul 04564, Republic of Korea; hanibalkms@hanmail.net; Tel.: +82-2-2260-7180

**Keywords:** intracranial hemorrhage, osteotomy, intraoral, coronal

## Abstract

Reduction malarplasty (RMP) is performed frequently in East Asia. Although various complications of RMP have been reported, intracranial hemorrhage has been described only rarely. Although intracranial hemorrhage is an extremely rare complication, it is a catastrophic event that should not occur in esthetic plastic surgery. In this review, I describe the occurrence and prevention of this serious complication through a detailed analysis of intracranial hemorrhage following RMP. Evaluation of RMP surgical techniques was performed. A search of PubMed and Google Scholar was conducted to identify studies reporting cerebral hemorrhage following RMP. The search strategy combined the terms “cosmetic surgery” OR “plastic surgery”, AND “cerebral hemorrhage”. The two major surgical approaches to RMP are the coronal and intraoral incisions. In the coronal approach, osteotomy is performed under direct visualization, which allows precise bone cutting. There is no risk of penetrating the intracranial cavity during RMP performed via the coronal approach. Two reported cases of intracranial hemorrhage occurred during RMP performed via the intraoral approach due to inappropriate instrument handling. There is a potential risk of injury to the middle cranial fossa by a reciprocating saw or chisel during osteotomy of the zygomatic body. When performing an osteotomy on a zygomatic body using a reciprocating saw in RMP via the intraoral approach, surgeons should avoid inserting the saw too deeply to prevent injury to the middle cranial fossa.

## 1. Introduction

Each year, thousands of patients undergo reduction malarplasty (RMP) in East Asia. Several complications associated with RMP have been reported. In an analysis of 14 articles including 3149 RMP cases, Myung et al. [1] reported complications such as transient sensory weakness (5.5%), drooping (2.8%), nonunion (2.2%), asymmetry (1.8%), restricted mouth opening (1.8%), uncontrollable bleeding (1.3%), and facial nerve injury (0.9%). According to their report [1], the overall complication rates associated with RMP were relatively low. Plastic surgeons have provided detailed descriptions of complications that occur after RMP [2]. These include facial asymmetry, facial sagging, inferior displacement of the zygoma, scarring alopecia, hyperostosis of the cheekbones, loosening of bony fixation, temporomandibular joint movement restriction, localized contour depression and irregularity, and osteolysis or resorption of the cheekbone. However, plastic surgeons may have limited awareness of intracranial hemorrhage following RMP, and relatively little attention has been given to this complication. Moreover, intracranial hemorrhage can result in serious neurological sequelae [3,4,5].

A thorough understanding of surgical anatomy and operative technique is essential to reduce complications after RMP. In this review, I examine several RMP techniques and describe the occurrence and prevention of this serious complication through a detailed analysis of intracranial hemorrhage following RMP.

## 2. Evaluation of RMP Surgical Techniques and Literature Search for Cases of Postoperative Cerebral Hemorrhage Following RMP Surgery

### 2.1. Evaluation of Surgical Techniques for RMP

To gain a precise understanding of the surgical techniques and intraoperative anatomical relationships associated with RMP, I searched for and reviewed relevant papers published in the PubMed database. I focused particularly on articles that provided detailed descriptions of the surgical procedures. To understand the three-dimensional spatial relationships of anatomical structures during surgery, it was necessary to make comparisons using a cadaver skull.

### 2.2. Literature Search for Cases of Postoperative Cerebral Hemorrhage Following RMP Surgery

A literature search was conducted in the PubMed database and Google Scholar (https://scholar.google.com) on 30 June 2026, without restrictions on language or publication time. Since searches on Google Scholar yielded different results even when using the same search terms, I performed the search 20 times. Abstracts of papers published by plastic surgery societies that hold annual meetings in South Korea, Taiwan, Japan, and China were not included in the analysis. Grey literature was not included in the literature search. The search strategy combined the terms “cosmetic surgery” OR “plastic surgery”, AND “cerebral hemorrhage” using multiple forms of Medical Subject Headings terms and Text words.

Two hundred and fifteen articles were obtained from the PubMed database (Supplementary Material S1). After the removal of duplicates and screening the abstracts and titles, the full texts of the remaining 51 articles (Supplementary Material S2) were analyzed. Among the papers retrieved from PubMed, there were no reported cases of cerebral hemorrhage following RMP.

A total of three cases of cerebral hemorrhage following RMP were identified in Google Scholar [3,4,5]. Kim et al. [3] reported an intracerebral hemorrhage in the temporal lobe caused by penetration of a reciprocating saw during RMP performed via the intraoral approach. Yim et al. [5] reported an epidural hemorrhage resulting from injury to the middle meningeal artery during RMP via the intraoral approach. The full text of Kim et al. [3] is available in Google Scholar; however, the article is written in Korean, with only the abstract and figure captions provided in English. The full text of Yim et al. [5] is written in English but is available only through the Airiti Library (airitilibrary.com). Lee et al. [4] described a case of subdural and subarachnoid hemorrhage after RMP; however, detailed clinical information regarding this complication was not provided. Intracranial hemorrhage after RMP is rare, and the limited number of published cases makes comprehensive analysis challenging. Therefore, in this review, I describe the occurrence and prevention of this serious complication through a detailed analysis of intracranial hemorrhage following RMP, with particular emphasis on surgical technique.

## 3. Association Between the Coronal Approach to RMP and Intracranial Hemorrhage

Several operative techniques for RMP have been described. The two major surgical approaches are the coronal and intraoral incisions [6,7,8]. In RMP, the choice of surgical technique by the plastic surgeon depends on three factors: patient preferences, the degree of zygomatic prominence, and the surgeon’s skill level [2]. The coronal approach provides a much wider operative field, enabling accurate cutting, and fixation of the malar complex. In contrast, the intraoral approach is more widely used because the longer incision required for the coronal approach is associated with prolonged operative time and a longer scalp scar. Furthermore, some patients decline a long scalp incision [9].

Zhang et al. [2] reported biomechanical changes in the zygomaticus and masseter muscles, as well as related complications, according to three RMP techniques. These considerations are important when plastic surgeons select a surgical method. However, intracranial complications should also be taken into account. The two reported cases of intracranial hemorrhage [3,5] occurred after RMP performed via the intraoral approach. The case reported by Lee et al. [4] did not include a detailed description of the surgical technique used. Based on these reports, intracranial hemorrhage related to surgical manipulation appears more likely to occur after the intraoral approach than after the coronal approach.

**Figure 1:**
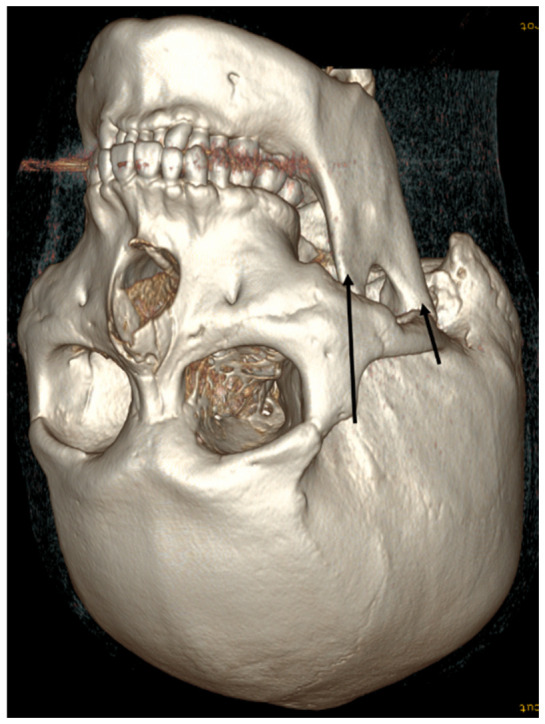
Surgical field of view according to the surgeon’s line of sight. Osteotomy in reduction malarplasty using the coronal approach under direct visualization (arrow: site and direction of osteotomy). The scalp flap is elevated, and subperiosteal dissection is performed to expose the zygoma. Osteotomies of both the zygomatic body and the zygomatic arch near the articular tubercle are performed under direct visualization. The tip of the reciprocating saw is directed from the cranial side toward the facial bone. For these two reasons, the risk of intracranial hemorrhage is negligible.

The coronal approach offers advantages for precise osteotomy because it provides a wide operative field for fixation of the malar complex [9,10]. RMP using the coronal approach is performed as follows [7,10]. An “M”-shaped scalp incision is made approximately 10 cm posterior to the hairline. Dissection in the temporal area is performed along the subtemporoparietal fascial plane. After identification of the bony margins of the zygomatic arch and body, the zygoma is fully exposed through subperiosteal dissection. The insertions of the temporalis fascia and the masseter muscle to the malar complex are preserved, except at the osteotomy sites. Medial (zygomatic body) and lateral (zygomatic arch near the articular tubercle) malar osteotomies are performed to mobilize the malar complex. The zygomatic arch near the articular tubercle is cut obliquely from posterior to anterior. RMP using the coronal approach allows accurate osteotomy and precise reduction in the maxillary bone within a wide surgical field. Therefore, this approach carries a very low risk of intracranial hemorrhage related to surgical manipulation for two reasons. First, the wide surgical field permits visualization of the full thickness of bone at the osteotomy site. Second, the tip of the reciprocating saw is oriented toward the facial surface rather than toward the cranial cavity, as shown in Figure 1. As long as the saw blade is aligned along the proper axis and direction, there is no risk of penetrating the intracranial cavity or skull.

## 4. RMP via the Intraoral Approach

For malar repositioning, a coronal incision has been proposed to allow accurate reconfiguration of the bone in its intended position. However, the coronal approach requires extensive dissection and is therefore more time-consuming than intraoral and preauricular incisions [11]. Consequently, various techniques for RMP have been introduced, including L-shaped osteotomy via the intraoral approach, limited preauricular incisional access, zygomatic arch reduction through multiple osteotomies, rotational techniques, facelift with malar repositioning, and boomerang osteotomy [12,13,14,15,16,17,18].

The intraoral incision alone, or in combination with preauricular and temporal incisions, is most commonly used because of its simplicity, strong fixation, and ability to achieve the desired degree of zygomatic reduction [15]. However, this approach is associated with several complications, including infraorbital nerve paresthesia, difficulty in creating symmetrical shapes, a limited maximum reduction volume occasionally resulting in under-correction, and cheek ptosis caused by extensive detachment of the masseter and zygomaticus major muscle insertions. Moreover, the intraoral approach is associated with additional technical challenges, such as difficult osteotomy and fixation due to limited exposure. The narrow operative field inherent to this approach makes it difficult to achieve a precise extent of osteotomy [9].

### 4.1. Lateral Osteotomy of the Zygomatic Arch near the Articular Tubercle

#### 4.1.1. Intraoral Approach Alone

**Figure 2:**
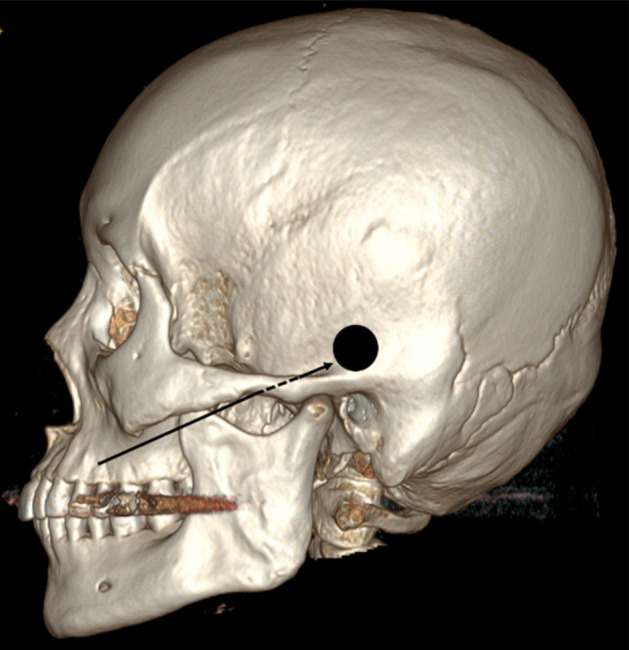
Surgical field of view according to the surgeon’s line of sight. Direction (arrow: direction of the saw) and location of osteotomy of the zygomatic arch near the articular tubercle during reduction malarplasty via the intraoral approach alone. This osteotomy is unlikely to damage the skull near the articular tubercle with a reciprocating saw because the cranial bone in this area is thick (black circle: potential area of skull injury; however, this region has substantial bony thickness).

The lateral osteotomy line of the zygomatic arch near the articular tubercle is created either as a greenstick fracture or as a full-thickness fracture via the intraoral approach [2,19,20]. The osteotomy of the zygomatic arch is performed posteromedially, approximately 1 cm anterior to the articular tubercle [19]. When the intraoral approach alone is used, osteotomy of the zygomatic arch near the articular tubercle poses little risk of skull fracture because the cranial bone in this region is relatively thick (Figure 2).

#### 4.1.2. Minimal Separate Incision in the Sideburn Area

**Figure 3:**
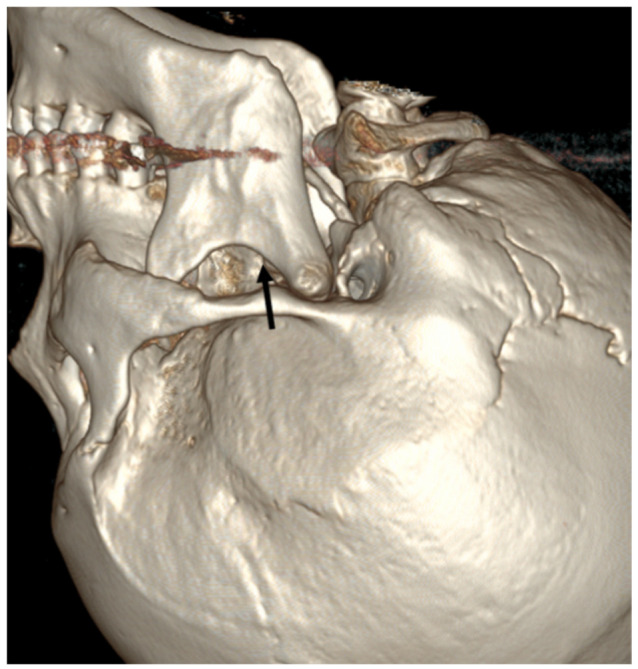
Surgical field of view according to the surgeon’s line of sight. Osteotomy position of zygomatic arch near the articular tubercle in reduction malarplasty using a preauricular incision under direct visualization. A minimal skin incision (15 mm in length) is made in the sideburn area. The zygomatic arch near the articular tubercle is exposed through dissection and then fractured using a small osteotome just in front of the articular tubercle. There is no risk of skull base injury during osteotomy (arrow: direction of the saw).

Ma et al. [18] and Cho [7] described another technique (Figure 3). A minimal separate incision is made in the sideburn area. The root of the zygomatic arch is exposed through dissection and then fractured with a small osteotome just anterior to the articular tubercle. Lee and Lee [21] presented a video demonstrating arch osteotomy using a preauricular incision. With this separate sideburn incision technique, osteotomy of the zygomatic arch is unlikely to cause skull base injury.

### 4.2. Medial Osteotomy

#### 4.2.1. L-Shaped Osteotomy of the Zygomatic Body

**Figure 4:**
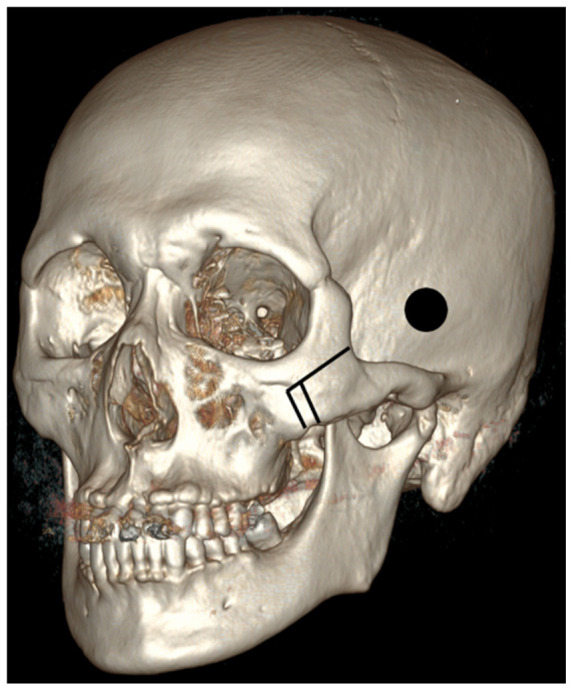
Surgical field of view according to the surgeon’s line of sight. Osteotomy position of L-shaped osteotomy in reduction malarplasty via the intraoral approach. The two vertical osteotomies do not pose a risk of middle cranial fossa damage. However, during the horizontal osteotomy, use of a reciprocating saw carries a potential risk of middle cranial fossa injury because the distal portion of the surgical field cannot be directly visualized (black circle: potential area of skull base injury).

An osteotomy of the zygomatic body performed via the intraoral approach carries a potential risk of injury to the middle cranial fossa. Wang et al. [22] described RMP using an L-shaped osteotomy of the zygomatic body (Figure 4). The degree of reduction is determined by the amount of bone removed between two parallel vertical osteotomy lines. An oblique osteotomy is made in the upper portion of the zygoma, approximately 5 mm inferior to the inferolateral margin of the orbit. Ma et al. [18] and Zhang et al. [2] also described RMP using an L-shaped osteotomy of the zygomatic body via the intraoral approach. In these techniques, two parallel vertical osteotomy lines are created on the anterior aspect of the zygomatic bone, near the inferior border of the zygomaticomaxillary suture. A horizontal osteotomy is then made from the lateral portion of the zygomaticofrontal suture toward the two vertical osteotomy lines [2,18]. Lee and Lee [21] presented an operative video demonstrating the L-shaped osteotomy of the zygomatic body. As shown in Video 2 [21], the tip of the reciprocating saw is not visible during osteotomy of the zygomatic body because of the narrow surgical field.

When performing an L-shaped osteotomy of the zygomatic body using a reciprocating saw or chisel, the vertical osteotomy does not pose a risk of skull base injury. Instead, it carries a potential risk of maxillary fracture. In contrast, during the horizontal osteotomy, the tip of the reciprocating saw may injure the middle cranial fossa.

#### 4.2.2. Linear Osteotomy of the Zygomatic Body

**Figure 5:**
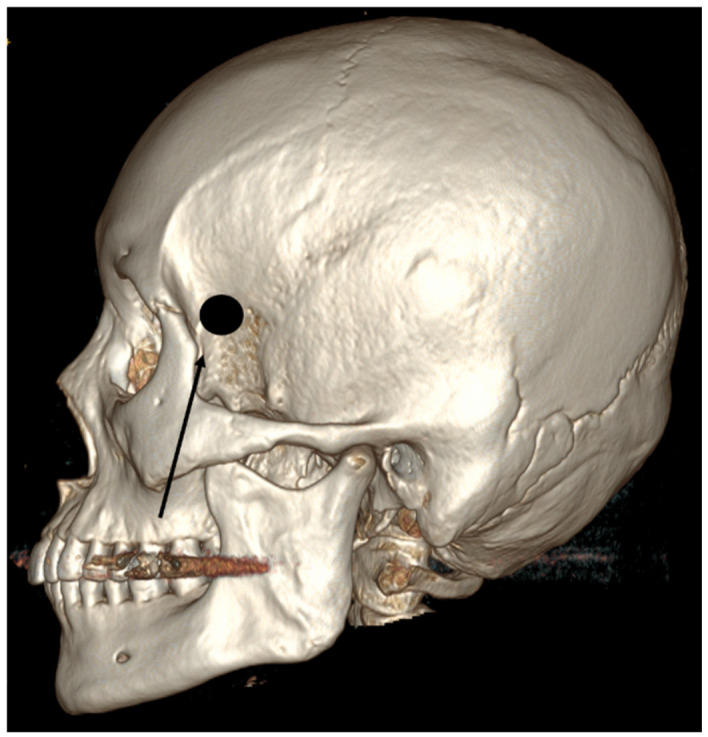
Surgical field of view according to the surgeon’s line of sight. Linear osteotomy orientation and position (arrow: direction of the saw) in reduction malarplasty using the intraoral approach. Use of a reciprocating saw in a blind surgical field may result in middle cranial fossa injury (black circle: potential area of skull base injury).

Lee et al. [20] and Zhang et al. [2] described a linear osteotomy of the zygomatic body. The medial osteotomy line is placed along the anterior border of the inner cortex of the zygomatic arch, approximately 0.5 cm posterior to the anterior border of the masseter attachment to the zygomatic bone. This medial osteotomy may injure the middle cranial fossa. Performing the osteotomy in this direction positions the tip of the reciprocating saw very close to the middle cranial fossa, thereby increasing the risk of middle cranial fossa injury (Figure 5).

Both intracranial hemorrhages reported by Kim et al. [3] and Yim et al. [5] may have resulted from injury to the middle cranial fossa during medial osteotomy of the zygomatic body. Blind manipulation in the deep portion of the zygoma via the intraoral approach, together with deep insertion of the reciprocating saw, may lead to injury of the middle cranial fossa. Because osteotomy in this region is performed without direct visualization, the surgeon’s experience is critical. The risk of these complications may be reduced if the surgeon accurately recognizes the position of the reciprocating saw tip through surgical experience and has a thorough understanding of anatomical landmarks. In particular, when performing osteotomy at the zygomatic body, it should be recognized that the reciprocating saw in a linear osteotomy is positioned closer to the middle cranial fossa than in an L-shaped osteotomy.

## 5. Possible Mechanisms of Intracranial Hemorrhage in the Reported Cases

Yim et al. [5] proposed two mechanisms for epidural hemorrhage after RMP performed via the intraoral approach. The first mechanism involves inappropriate manipulation of a reciprocating saw and other instruments during medial osteotomy of the zygomatic arch. The second mechanism suggests that force applied to the temporal bone and bony spicules while elevating the fractured zygomatic arch may result in tearing of the middle meningeal artery. The former mechanism appears to be the more plausible explanation for epidural hemorrhage secondary to middle meningeal artery injury. Elevation of both the zygomatic body and arch is unlikely to transmit sufficient force to the middle cranial fossa, through which the middle meningeal artery passes. Instead, such elevation would more likely cause injury to the orbit or sphenoid bone. In Figure 1 of Yim et al.’s report [5], no apparent injury to the orbit or sphenoid bone on the left side of the skull is observed.

**Figure 6:**
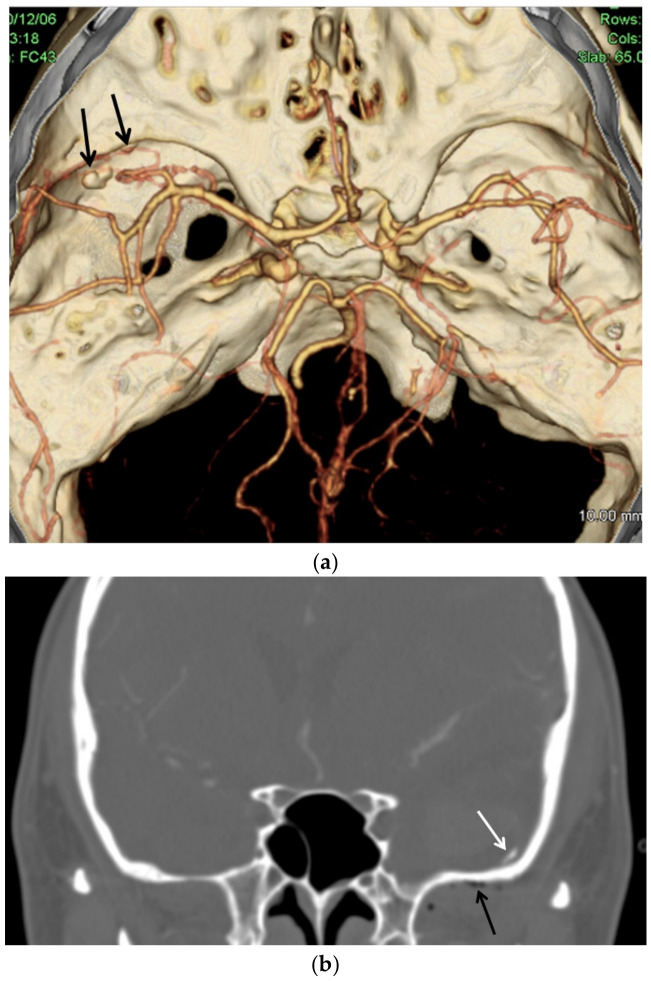
Brain computed tomography angiography (**a**) demonstrates bony abnormalities presumed to have been caused by improper use of a reciprocating saw during surgery (black arrows: bony abnormalities). Coronal computed tomography (**b**) shows a bony fragment (white arrow) within the intracranial cavity and an air bubble (black arrow) in the extracranial region of the left middle cranial fossa. (These images correspond to Figure 6 in Kim et al. [3]. Permission to reproduce the images was obtained from the Editor-in-Chief of the *Korean Journal of Neurotrauma*).

**Figure 7:**
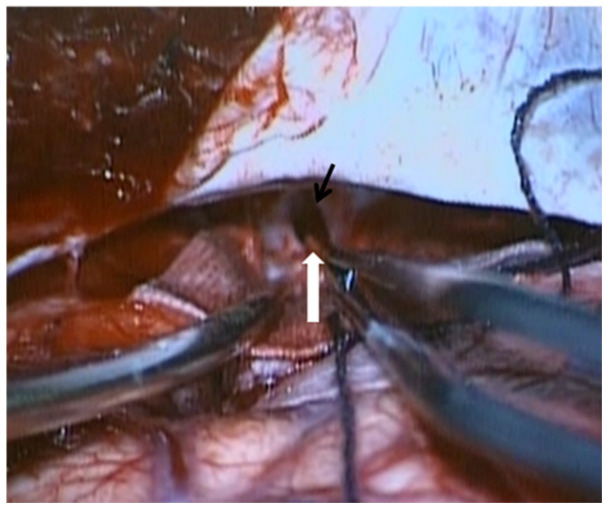
Intraoperative photograph obtained after evacuation of the intracerebral hemorrhage shows a dural defect (black arrow) in the middle cranial fossa and a bony fragment (white arrow) within the intracranial cavity. (This image corresponds to Figure 4 in Kim et al. [3]. Permission to reproduce the image was obtained from the Editor-in-Chief of the *Korean Journal of Neurotrauma*).

Kim et al. [3] reported that a temporal lobe hematoma developed following aggressive instrument manipulation (Figure 6). During craniotomy for evacuation of the intracranial hemorrhage, a dural defect in the middle cranial fossa, a middle cranial fossa bony defect, and a bony fragment within the intracranial cavity were identified (Figure 7). Kim et al. [3] suggested that the osteotomy procedure of the zygomatic body may have resulted in intracerebral hemorrhage.

However, Yim et al. [5] and Kim et al. [3] did not describe the detailed surgical technique used for RMP. During osteotomy of the zygomatic body, excessive insertion of the reciprocating saw may result in injury to the middle cranial fossa (Figure 6), followed by the development of epidural or intracerebral hemorrhage. Lee et al. [4] also reported subdural and subarachnoid hemorrhage in a 24-year-old female patient after RMP and submental liposuction. The patient subsequently progressed to brain death. Although imaging and autopsy findings were not available, the mechanism of cerebral hemorrhage in this case is presumed to be similar to that in the two previously described cases. Spontaneous subdural and subarachnoid hemorrhage are rare in young patients without coagulation disorders or other underlying diseases.

Preoperative measurement of the distance between the zygomatic body and the middle cranial fossa using three-dimensional computed tomography may help prevent such complications. When using a reciprocating saw via the intraoral approach, if the tip is perceived to contact bone unexpectedly, the surgeon should immediately discontinue use of the saw and reassess the anatomical position.

## 6. Surgical Treatment of Intracranial Hemorrhage After RMP

Intracranial hemorrhage occurring after RMP performed via the intraoral approach may present as an epidural hemorrhage originating from the middle cranial fossa, an intracerebral hemorrhage in the temporal lobe, or a subdural hemorrhage. In such cases, surgical management for hematoma evacuation is similar to that for cerebral hemorrhage caused by other forms of trauma. Craniotomy is required for the following reasons. When stereotactic evacuation of an intracerebral hemorrhage is attempted through a burr hole, there is a risk of rebleeding and inadequate hemorrhage control. In addition, craniotomy is mandatory in cases of epidural or subdural hemorrhage. Although the RMP procedure is performed intraorally, the risk of ascending infection is low. If a dural defect is identified in the middle cranial fossa, cerebrospinal fluid leakage may occur. Therefore, repair of the dural defect is necessary using tissue adhesives such as Greenplast (Greenplast, GC Biopharma, Yongin-si, Republic of Korea), artificial dura mater, or autologous fascia.

Sequelae were reported in all three cases [3,4,5] described in the literature, and one of the three cases resulted in death. Therefore, preventive measures to avoid intracranial hemorrhage are of paramount importance.

## 7. Conclusions

Each surgical technique in RMP has distinct advantages and disadvantages. The conventional coronal approach is unlikely to cause intracranial hemorrhage because the orientation of the reciprocating saw does not place the skull base at risk. In contrast, when performing osteotomy of the zygomatic body via the intraoral approach, there is a risk of injury to the middle cranial fossa by the tip of the reciprocating saw, particularly when the saw is inserted excessively deeply during osteotomy of the malar complex.

## Data Availability

No new data were created or analyzed in this study. Data sharing is not applicable to this article.

## Supplementary Materials

The following supporting information can be downloaded at: https://www.mdpi.com/article/10.3390/jcm15145609/s1, Supplementary Material S1 is a paper list retrieved from the PubMed database. Supplementary Material S2 is papers that underwent full-text evaluation after screening of duplicate articles, titles, and abstracts.

## Abbreviations

The following abbreviations are used in this manuscript:

RMP	Reduction malarplasty
