# Supplementary material for: Intracranial Hemorrhage After Reduction Malarplasty: A Narrative Review Focusing on Surgical Technique
Source: J Clin Med. 2026 Jul 17;15(14):5609. doi: 10.3390/jcm15145609 (PMC13413184; doi:10.3390/jcm15145609)
Supplement: Supplementary file 1 [file jcm-15-05609-s001.zip › Supplementary Material S1.pdf]

Search result from Pubmed database (215 articles)

1: Zhang J, Liu H, Liu Y, Liu S, He Z, Chen G, Luo E. A Systematic Review and Meta-Analysis of Complications among Various Reduction Malarplasty. Aesthetic Plast Surg. 2023 Jun;47(3):1018-1038. doi: 10.1007/s00266-022-03104-1. Epub 2022 Oct 19. Erratum in: Aesthetic Plast Surg. 2023 Dec;47(6):2909. doi: 10.1007/s00266-023-03454-4. PMID: 36261745.

2: Jin Q, He Y, Yu B, Liu J, Fu X, Xu S, Qiao J, Chen Y, Gui L. The Spatial Relationship Between Coronoid Process and Zygomatic Complex After Reduction Malarplasty. Aesthetic Plast Surg. 2024 Apr;48(8):1529-1536. doi: 10.1007/s00266-024-03897-3. Epub 2024 Feb 29. PMID: 38424305.

3: Ha SH, Jung S, Choi JY. Reduction Malarplasty Using Customized Surgical Stent Based on 3D Virtual Surgery, CAD/CAM, and 3D Printing Technology: Case Series. J Craniofac Surg. 2022 Jul-Aug 01;33(5):1578-1582. doi: 10.1097/SCS.0000000000008430. Epub 2021 Dec 14. PMID: 34907946.

4: Fu X, Mao X, Gui L, Niu F, Liu J, Jia Q, Chen Y. Zygomatic Nonunion: A Misunderstood Complication of Reduction Malarplasty. J Craniofac Surg. 2019 May/Jun;30(3):e207-e209. doi: 10.1097/SCS.0000000000004665. PMID: 30444773.

5: Wang Y, Hsu Y, Chen G, Bi D, Wang Y, Li J. The importance of the mortice and tenon joint on the zygomatic arch in reduction malarplasty-a retrospectivestudy. J Craniomaxillofac Surg. 2022 Apr;50(4):316-321. doi:10.1016/j.jcms.2021.12.012. Epub 2022 Jan 15. PMID: 35125285.

6: Lee JS, Lee JW, Yang JD, Chung HY, Cho BC, Choi KY. A rare complication of reduction malarplasty. Aesthetic Plast Surg. 2015 Apr;39(2):240-2. doi: 10.1007/s00266-014-0441-0. Epub 2015 Jan 22. PMID: 25608911.

7: Myung Y, Kwon H, Lee SW, Baek RM. Postoperative Complications Associated With Reduction Malarplasty via Intraoral Approach: A Meta Analysis. Ann Plast Surg. 2017 Apr;78(4):371-378. doi: 10.1097/SAP.0000000000000913. PMID: 27801697.

8: Qiao C, Xu J, Shi Z, Li J, Miao J, Yan S, Qu Y, Wu G. Combined partial coronoidectomy and reduction malarplasty versus reduction malarplasty alone in patients with elongated coronoid processes: A comparative retrospective study. J Craniomaxillofac Surg. 2026 Aug;54(8):104600. doi: 10.1016/j.jcms.2026.104600. Epub 2026 May 24. PMID: 42177903.

9: Moon JH, Lee GH. Fatal Meningitis Following Reduction Malarplasty: A Previously Unreported Complication. J Craniofac Surg. 2026 Jun 1. doi: 10.1097/SCS.0000000000013016. Online ahead of print

10.1097/SCS.0000000000013016. Epub ahead of print. PMID: 42223980.10: Rodman R. Cosmetic bone contouring. Curr Opin Otolaryngol Head Neck Surg. 2017 Aug;25(4):337-340. doi: 10.1097/MOO.0000000000000370. PMID: 28504986.

11: Zhang J, Liu H, Liu Y, Liu S, He Z, Chen G, Luo E. Correction: A Systematic Review and Meta-Analysis of Complications Among Various Reduction Malarplasty. Aesthetic Plast Surg. 2023 Dec;47(6):2909. doi: 10.1007/s00266-023-03454-4. Erratum for: Aesthetic Plast Surg. 2023 Jun;47(3):1018-1038. doi: 10.1007/s00266-022-03104-1. PMID: 37353599.

12: Gao ZW, Wang WG, Zeng G, Lu H, Ma HH. A modified reduction malarplasty utilizing 2 oblique osteotomies for prominent zygomatic body and arch. J Craniofac Surg. 2013 May;24(3):812-7. doi: 10.1097/SCS.0b013e31828dcd88. PMID: 23714886.

13: Chung S, Park S. Lowering Lateral Canthoplasty and Orbital Rim Shaving: An Ignored but Necessary Procedure for Maximizing the Effect of Reduction Malarplasty in Asians. Aesthetic Plast Surg. 2019 Jun;43(3):686-694. doi: 10.1007/s00266-019-01342-4. Epub 2019 Mar 21. PMID: 30903250.

14: Hsiao CW, Hsiao KY, Shen YD, Zavala A, Hsiao YW. Easy and Safe Simultaneous Zygoma Reduction and Facelift by Single Incision Through Subcutaneous Approach. J Plast Reconstr Aesthet Surg. 2022 Sep;75(9):3513-3520. doi:10.1016/j.bjps.2022.05.002. Epub 2022 Jun 15. PMID: 35821011.

15: Gao W, Qiu Y, Zou Y, Qiao C, Chang L, Jin Y, Chen H, Lin X. Reduction Malarplasty With Subperiosteal Lift Via a Single Limited Temporal Incision. Ann Plast Surg. 2021 Mar 1;86(3S Suppl 2):S194-S198. doi: 10.1097/SAP.0000000000002644. PMID: 33651014.

16: Han MD, Kwon TG. Zygoma and Mandibular Angle Reduction: Contouring Surgery to Correct the Square Face in Asians. Oral Maxillofac Surg Clin North Am. 2023 Feb;35(1):83-96. doi: 10.1016/j.coms.2022.06.003. Epub 2022 Nov 3. PMID: 36336603.

17: Tan W, Niu F, Yu B, Gui L. Feasibility of absorbable plates and screws for fixation in reduction malarplasty with L-shaped osteotomy. J Craniofac Surg. 2011 Mar;22(2):546-50. doi: 10.1097/SCS.0b013e318208bb41. PMID: 21403525.

18: Mu X. Experience in East Asian facial recontouring: reduction malarplasty and mandibular reshaping. Arch Facial Plast Surg. 2010 Jul-Aug;12(4):222-9. doi: 10.1001/archfacial.2010.48. PMID: 20644225.

19: Choi BK, Seo JY, Seo HJ, Choi SJ, Lee JW, Kim MW, Nam SB, Bae YC. Analysis and Guidelines for Revisional Malarplasty; Most Common Facial Skeletal Contouring Surgery. J Craniofac Surg. 2022 Sep 1;33(6):1674-1678. doi: 10.1097/SCS.0000000000008403. Epub 2021 Dec 7. PMID: 34879016.

20: Dong G, Teng L, Lu J, Huang Y. Application of the Bracing System in Reduction Malarplasty in Asian Population. Aesthetic Plast Surg. 2020 Feb;44(1):114-121. doi: 10.1007/s00266-019-01532-0. Epub 2019 Nov 7. PMID: 31701202.

21: Kim JH, Lee HY, Jeong JY, Kim TK. Radiologic Analysis of Malar Arch Movement in Reduction Malarplasty Without Bony Resection. J Craniofac Surg. 2021 Jun 1;32(4):1307-1310. doi: 10.1097/SCS.0000000000007168. PMID: 33177417.

22: Zou C, Wang JQ, Liu JF, Niu F, Chen Y, Wang M, Gui L. Reduction Malarplasty With Face-Lift for

Older Asians With Prominent Zygoma. Ann Plast Surg. 2016 Aug;77(2):141-4. doi: 10.1097/SAP.0000000000000586. PMID: 26207549.

23: Hwang CH, Lee MC. Reduction malarplasty using a zygomatic arch-lifting technique. J Plast Reconstr Aesthet Surg. 2016 Jun;69(6):809-818. doi: 10.1016/j.bjps.2016.03.004. Epub 2016 Mar 23. PMID: 27084574.

24: Yang HW, Hong JJ, Koo YT. Reduction Malarplasty that Uses Malar Setback Without Resection of Malar Body Strip. Aesthetic Plast Surg. 2017 Aug;41(4):910-918. doi: 10.1007/s00266-017-0879-y. Epub 2017 May 23. PMID: 28536928.

25: Zhang Y, Tang M, Jin R, Zhang Y, Zhang Y, Wei M, Qi Z. Comparison of three techniques of reduction malarplasty in zygomaticus and massateric biomechanical changes and relevant complications. Ann Plast Surg. 2014 Aug;73(2):131-6. doi: 10.1097/SAP.0b013e318273f81f. PMID: 23407255.

26: Gao B, Yuan Y, Li K, Li Z, Yu L. Facial Contour Rejuvenation by Reduction Malarplasty Combined With Second-Stage Fat Grafting. J Craniofac Surg. 2021 Jan-Feb 01;32(1):179-183. doi: 10.1097/SCS.0000000000007043. PMID: 33196618.

27: Cho J, Kwon JS, Lee UL. Occlusion-Fit Three-Dimensional-Printed Zygoma Repositioner. J Craniofac Surg. 2018 May;29(3):731-732. doi: 10.1097/SCS.0000000000004315. PMID: 29419598.

28: Baek RM, Kim J, Lee SW. Revision reduction malarplasty with coronal approach. J Plast Reconstr Aesthet Surg. 2010 Dec;63(12):2018-24. doi: 10.1016/j.bjps.2010.01.007. Epub 2010 Feb 13. PMID: 20153991.

29: Ge H, Wang Y, Gao H, Sun X, Wu Y, Li J. The accuracy of virtual surgical planning assisted management for L-shaped reduction malarplasty: A retrospective study. J Craniomaxillofac Surg. 2024 Mar;52(3):363-368. doi: 10.1016/j.jcms.2024.01.016. Epub 2024 Jan 19. PMID: 38278743.

30: Jiang T, Fang B, Yu Z, Cao D. Hoarseness and arytenoid dislocation: A rare complication after facial bony contouring surgery. J Plast Reconstr Aesthet Surg. 2023 Sep;84:432-438. doi: 10.1016/j.bjps.2023.06.014. Epub 2023 Jun 10. PMID: 37413735.

31: Kim JJ, Lee EY, Seok H, Kang JY. An improved technique for zygoma reduction malarplasty. J Craniomaxillofac Surg. 2018 Apr;46(4):654-659. doi: 10.1016/j.jcms.2018.01.010. Epub 2018 Feb 2. PMID: 29519577.

32: Kim JW, Hwang W. Optimal Fixation Location in Intraoral Reduction Malarplasty Using an L-Shaped Osteotomy. J Craniofac Surg. 2019 Nov-Dec;30(8):2490-2492. doi: 10.1097/SCS.0000000000005860. PMID: 31469740.

33: Kim T, Baek SH, Choi JY. Reduction malarplasty according to esthetic facial unit analysis: retrospective clinical study of 23 cases. J Oral Maxillofac Surg. 2014 Aug;72(8):1565-78. doi: 10.1016/j.joms.2014.02.029. Epub 2014 Feb 25. PMID: 24768421.

34: Li Q, Gao B, Li K, Xie F, Zhu H, Yu LG. A Novel Technique for Reduction Malarplasty by Inward Displacement of Infractured Zygomatic Arch Without Fixation. J Oral Maxillofac Surg. 2017 Dec;75(12):2658-2666. doi: 10.1016/j.joms.2017.06.022. Epub 2017 Jun 24. PMID: 28710911.

35: Lin LX, Yuan JL, Wang YT, Huang Y, Wang P, Wang XM. A New Infracture Technique for Reduction Malarplasty with an L-Shaped Osteotomy Line. Med Sci Monit. 2015 Jul 6;21:1949-54. doi: 10.12659/MSM.893503. PMID: 26145181; PMCID: PMC4501642.

36: Hwang K. Lateral rectus muscle injury, orbital fracture, mouth locking, and facial palsy resulting from reduction malarplasty. J Craniofac Surg. 2011 Jan;22(1):151-4. doi: 10.1097/SCS.0b013e3181f6fa0d. PMID: 21187757.

37: Qiao C, Xu J, Miao J, Shi Z, Yan K, Yan S, Qu Y, Wu G. Enhanced Midface Contouring:

Simultaneous Zygomatic Reduction and Partial Coronoidectomy for Optimal Aesthetic and Functional Outcomes. Aesthetic Plast Surg. 2025 Oct;49(20):5725-5733. doi: 10.1007/s00266-025-05158-3. Epub 2025 Aug 13. PMID: 40801932.

38: Lee JS, Kim EH, Lee SH. Endoscopically assisted malarplasty: L-rotation technique. J Stomatol Oral Maxillofac Surg. 2021 Jun;122(3):229-234. doi: 10.1016/j.jormas.2020.07.011. Epub 2020 Aug 15. PMID: 32810601.

39: Subu M, Paeng JY. Traumatic Bony Ankylosis of Temporomandibular Joint as a Complication After Reduction Malarplasty. J Craniofac Surg. 2018 Jun;29(4):e416-e417. doi: 10.1097/SCS.0000000000004421. PMID: 29554062.

40: Al-Watary MQ, Hao J, He Y, Song L, Gao H, Alkebsi K, Elayah SA, Ye B, Li J. Evaluation of Different Fixation Methods Combinations After L-Shaped Osteotomy Reduction Malarplasty: An In Vitro Biomechanical Study. Aesthetic Plast Surg. 2024 Aug;48(16):3170-3179. doi: 10.1007/s00266-024-03960-z. Epub 2024 Mar 22. PMID: 38519573.

41: Kotha VS, Kanuri A, Mandelbaum M, Lakhiani C, Hung RW, Wang J, Rashid W, Chao JW. Simultaneous Zygomatic Osteotomies With Reduction Mandibuloplasty – An Approach to Mid- and Lower-Facial Feminization in the Transfeminine Patient. J Craniofac Surg. 2022 Jul-Aug 01;33(5):1569-1573. doi: 10.1097/SCS.0000000000008386. Epub 2021 Dec 1. PMID: 34855635.

42: Lee YH, Lee SW. Zygomatic nonunion after reduction malarplasty. J Craniofac Surg. 2009 May;20(3):849-52. doi: 10.1097/scs.0b013e3181a2f040. PMID: 19480043.

43: Zhou J, Zheng H, Qi Z, Jin X. Unexpected Total Zygomatic Arch Bone Resorption Induced by Reduction Malarplasty. J Craniofac Surg. 2019 Oct;30(7):2211-2213. doi: 10.1097/SCS.0000000000005799. PMID: 31369506.

44: Zhang Q, Chang C, Meng Z, Huang J, Guo J, Ge Z. Novel treatment of revision malarplasty with

piezosurgery: A case report. Medicine (Baltimore). 2020 Oct 9;99(41):e22529. doi: 10.1097/MD.0000000000022529. PMID: 33031295; PMCID: PMC7544381.

45: Hwang JH, Kim ES, Kim KS. Dental pulp necrosis as a rare complication of reduction malarplasty. J Craniofac Surg. 2007 Jul;18(4):945-8. doi: 10.1097/scs.0b013e3180690072. PMID: 17667692.

46: Yang X, Mu X, Yu Z, Gu Q, Cao D, Yu D, Wei M, Chang T. Compared study of Asian reduction malarplasty: wedge-section osteotomy versus conventional procedures. J Craniofac Surg. 2009 Sep;20 Suppl 2:1856-61. doi: 10.1097/SCS.0b013e3181b6c65f. PMID: 19816365.

47: Baek RM, Kim J, Kim BK. Three-dimensional assessment of zygomatic malunion using computed tomography in patients with cheek ptosis caused by reduction malarplasty. J Plast Reconstr Aesthet Surg. 2012 Apr;65(4):448-55. doi: 10.1016/j.bjps.2011.10.019. Epub 2011 Nov 17. PMID: 22099146.

48: Kim DH, Jeon EJ, Jang SY, Kim HJ. Maxillary sinusitis after reduction malarplasty. Ann Plast Surg. 2011 Dec;67(6):577-8. doi: 10.1097/SAP.0b013e31820b429a. PMID: 21407053.

49: Kim TG, Cho YK. A New Double Trapezoid-Shaped Osteotomy for Reduction Malarplasty. J Craniofac Surg. 2016 Jan;27(1):87-93. doi: 10.1097/SCS.0000000000002268. PMID: 26703047.

50: Choi BK, Lee KT, Oh KS, Yang EJ. Preservation of the deep facial vein in reduction malarplasty. J Craniofac Surg. 2012 May;23(3):e254-7. doi: 10.1097/SCS.0b013e3182518845. PMID: 22627450.

51: Choung JW. Rotation technique of reduction malar plasty. J Craniofac Surg. 2015 Jan;26(1):238-9. doi: 10.1097/SCS.0000000000001199. PMID: 25569399; PMCID: PMC4297216.

52: Yuan J, Cho MY, Zhang Y, Qi ZL, Wei M. Influence of the maxillary sinus exposure in reduction malarplasty with an L-shaped osteotomy. J Craniofac Surg. 2011 Sep;22(5):1788-90. doi: 10.1097/SCS.0b013e31822e776b. PMID: 21959433.

53: Hwang CH, Lee MC. Revision malarplasty guided by strategic categorization. J Plast Reconstr Aesthet Surg. 2019 Feb;72(2):322-334. doi: 10.1016/j.bjps.2018.10.024. Epub 2018 Nov 13. PMID: 30514620.

54: Shan L, Liu DL, Yuan Q. [Anatomic reduction of midfacial sag after intraoral reduction malarplasty]. Nan Fang Yi Ke Da Xue Xue Bao. 2007 Mar;27(3):310-1. Chinese. PMID: 17425980.

55: Lee SB, Lee JH, Min HJ. External radiopaque marking of Gillies posterior zygomatic arch osteotomy in reduction malarplasty. J Craniomaxillofac Surg. 2016 Jul;44(7):783-8. doi: 10.1016/j.jcms.2016.04.019. Epub 2016 Apr 22. PMID: 27211348.

56: Kim JH, Yoon SM, Choi HJ. Iatrogenic arteriovenous fistula of the superficial temporal artery after reduction malarplasty. J Craniofac Surg. 2015 Jan;26(1):e50-1. doi: 10.1097/SCS.0000000000001312. PMID: 25569414.

57: Kim YH, Seul JH. Reduction malarplasty through an intraoral incision: a new method. Plast Reconstr Surg. 2000 Dec;106(7):1514-9. doi: 10.1097/00006534-200012000-00011. PMID: 11129179.

58: Wang T, Gui L, Tang X, Liu J, Yu D, Peng Z, Song B, Song T, Niu F, Yu B. Reduction malarplasty with a new L-shaped osteotomy through an intraoral approach: retrospective study of 418 cases. Plast Reconstr Surg. 2009 Oct;124(4):1245-1253. doi: 10.1097/PRS.0b013e31819e6562. PMID: 19935309.

59: Kim HI, Roh SG, Lee NH, Yang KM. Giant maxillary mucocele occurring after reduction malarplasty. J Craniofac Surg. 2012 Mar;23(2):e123-4. doi: 10.1097/SCS.0b013e31824cda4a. PMID: 22446443.

60: Mahatumarat C, Rojvachiranonda N. Reduction malarplasty without external incision: a simple technique. Aesthetic Plast Surg. 2003 May-Jun;27(3):167-71. doi: 10.1007/s00266-003-0083-0. Epub 2003 Aug 21. PMID: 12925859.

61: Song IS, Choi J, Baik UB, Ryu JJ, Lim JW, Choi YJ, Lee UL. Recovery pattern following bimaxillary orthognathic surgery: Differences between sexes. J Craniomaxillofac Surg. 2019 Jan;47(1):138-142. doi: 10.1016/j.jcms.2018.11.003. Epub 2018 Nov 10. PMID: 30501927.

62: Lee TS. Standardization of surgical techniques used in facial bone contouring. J Plast Reconstr Aesthet Surg. 2015 Dec;68(12):1694-700. doi: 10.1016/j.bjps.2015.08.010. Epub 2015 Aug 19. PMID: 26346781.

63: Shim BK, Shin HS, Nam SM, Kim YB. Real-time navigation-assisted orthognathic surgery. J Craniofac Surg. 2013 Jan;24(1):221-5. doi: 10.1097/SCS.0b013e318267bb76. PMID: 23348289.

64: Zhou J, Qi Z, Jin X. Simultaneous Surgery for Contouring the Prominent Zygoma and Mandibular Angles With Facelift in Middle-Aged Patients. J Craniofac Surg. 2020 Mar/Apr;31(2):448-452. doi: 10.1097/SCS.0000000000006227. PMID: 31977701.

65: Younis H, Zhou Z, Sun X, Ge H, Wang Y, Li J. Middle and lower-facial feminization surgery in East Asian transgender and cisgender women: Surgical techniques and outcomes. J Plast Reconstr Aesthet Surg. 2025 May;104:215-224. doi: 10.1016/j.bjps.2025.03.024. Epub 2025 Mar 12. PMID: 40154114.

66: Hong SE, Liu SY, Kim JT, Lee JH. Intraoral zygoma reduction using L-shaped osteotomy. J Craniofac Surg. 2014 May;25(3):758-61. doi: 10.1097/SCS.0000000000000759. PMID: 24657982; PMCID: PMC4025629.

67: He Y, Wang Y, Al-Watary MQ, Wang Y, Wu Y, Li X, Ye B, Li J. The L-Shaped Zygomatic Reduction with Oblique or Vertical Resection: Which One Is the Optimal Choice? Plast Reconstr Surg. 2025 Jan 1;155(1):26e-34e. doi: 10.1097/PRS.0000000000011396. Epub 2024 Mar 12. PMID: 38470996.

68: Liao L, Hsu Y, Hu J, Li X, Li H, Li J. Correction of asymmetric facial deformity by contouring: indications and outcomes. J Craniofac Surg. 2015 Mar;26(2):e94-8. doi:

10.1097/SCS.0000000000001026. PMID: 25643344.

69: Zou C, Niu F, Liu JF, Yu B, Chen Y, Wang M, Gui L. Application of Computer Techniques in Correcting Mild Zygomatic Assymetry With Unilateral Reduction Malarplasty. J Craniofac Surg. 2015 Sep;26(6):2002-4. doi: 10.1097/SCS.0000000000001894. PMID: 26359703.

70: Yoo HS, Byun IH, Ahn DK, Lee JH, Yoo WM. Multiplane Facelift Following Facial Bone Contouring for Enhanced Mobilization and Rejuvenation. J Craniofac Surg. 2020 Oct;31(7):1962-1966. doi: 10.1097/SCS.0000000000006516. PMID: 32398619.

71: Rhee DY, Kim SH, Shin DH, Uhm KI, Song WC, Koh KS, Choi HG. Lateral facial contouring via a single preauricular incision. J Plast Reconstr Aesthet Surg. 2012 Aug;65(8):e205-12. doi: 10.1016/j.bjps.2012.03.018. Epub 2012 Apr 9. PMID: 22495013.

72: Liu D, Huang J, Shan L, Wang J. Intraoral curved ostectomy for prominent mandibular angle by grinding, contiguous drilling, and chiseling. J Craniofac Surg. 2011 Nov;22(6):2109-13. doi: 10.1097/SCS.0b013e318232a58a. PMID: 22067875.

73: Shao Z, Xie Y, Yu B, Liu L, Du T. A new assisted fixation technique to prevent zygoma displacement in malar reduction. Aesthetic Plast Surg. 2013 Aug;37(4):692-6. doi: 10.1007/s00266-012-0033-9. Epub 2013 Jan 8. PMID: 23296763.

74: Young VL, Schuster RH, Harris LW. Intracerebral hematoma complicating split calvarial bone-graft harvesting. Plast Reconstr Surg. 1990 Oct;86(4):763-5. doi: 10.1097/00006534-199010000-00029. PMID: 2217594.

75: Charoonsmith T, Suwanwela C. Frontoethmoidal encephalomeningocele with special reference to plastic reconstruction. Clin Plast Surg. 1974 Jan;1(1):27-47. PMID: 4426155.

76: Yang C, Zhou L, Gao X, Chen B, Tu J, Sun H, Liu X, He J, Liu J, Yuan Q. Neuroprotective effects of bone marrow stem cells overexpressing glial cell line-derived neurotrophic factor on rats with intracerebral hemorrhage and neurons exposed to hypoxia/reoxygenation. Neurosurgery. 2011 Mar;68(3):691-704. doi: 10.1227/NEU.0b013e3182098a8a. PMID: 21311297.

77: Eufinger H, Wehmöller M, Machtens E, Heuser L, Harders A, Kruse D. Reconstruction of craniofacial bone defects with individual alloplastic implants based on CAD/CAM-manipulated CT-data. J Craniomaxillofac Surg. 1995 Jun;23(3):175-81. doi: 10.1016/s1010-5182(05)80007-1. PMID: 7673445.

78: Nguyen HS, Janich K, Sharma A, Patel M, Mueller W. To Retain or Remove the Bone Flap During Evacuation of Acute Subdural Hematoma: Factors Associated with Perioperative Brain Edema. World Neurosurg. 2016 Nov;95:85-90. doi: 10.1016/j.wneu.2016.07.067. Epub 2016 Jul 28. PMID: 27476687.

79: Schefold JC, Messmer AS, Wenger S, Müller L, von Haehling S, Doehner W, McPhee JS, Fux M, Rösler KM, Scheidegger O, Olariu R, Z'Graggen W, Rezzi S, Grathwohl D, Konz T, Takala J, Cuenoud B, Jakob SM. Nutrient pattern analysis in critically ill patients using Omics technology (NAChO) - Study protocol for a prospective observational study. Medicine (Baltimore). 2019 Jan;98(1):e13937.

doi: 10.1097/MD.0000000000013937. PMID: 30608424; PMCID: PMC6344160.

80: Holmgren K, Fjellborg M, Nilsson RF, Lindvall P, Corell A, Harba D, Fletcher-Sandersjöö A, Birgersson U, Sæmundsson B, Ågren R, Kihlström Burenstam Linder L, Sundblom J, Ryttlefors M, Svedung Wettervik T. Cranioplasty outcomes after decompressive craniectomy: a near-nationwide population-based study based on 15 years of cranial reconstructions in Sweden. J Neurosurg. 2026 Jan16;144(4):857-867. doi: 10.3171/2025.8.JNS25925. PMID: 41569675.

81: Akhtar MU, Akram M, Ahmed TM, Bhatti AM. Superficial temporal artery – middle cerebral artery bypass for internal carotid artery petrous aneurysm: A case report. J Pak Med Assoc. 2017 Jan;67(1):128-130. PMID: 28065970.

82: Rogers AC, Bourke M, Galbraith AS, Ryan AG, Cross KS, McMonagle MP. Mycotic Aneurysm of the Extracranial Internal Carotid Artery, Resect and Ligate or Reconstruct? Ann Vasc Surg. 2016 Aug;35:203.e5-203.e10. doi: 10.1016/j.avsg.2016.01.043. Epub 2016 May 26. PMID: 27238997.

83: Hashimoto N. Microsurgery for cerebral arteriovenous malformations: a dissection technique and its theoretical implications. Neurosurgery. 2001 Jun;48(6):1278-81. doi: 10.1097/00006123-200106000-00018. PMID: 11383730.

84: Ozerdem OR, Anlatici R, Sen O, Yildirim T, Bircan S, Aydin M. Prefabricated galeal flap based on superficial temporal and posterior auricular vessels. Plast Reconstr Surg. 2003 Jun;111(7):2166-75. doi: 10.1097/01.PRS.0000060109.58552.B2. PMID: 12794456.

85: Derdyn C, Persing JA, Broaddus WC, Delashaw JB, Jane J, Levine PA, Torner J. Craniofacial trauma: an assessment of risk related to timing of surgery. Plast Reconstr Surg. 1990 Aug;86(2):238-45; discussion 246-7. PMID: 2367573.

86: Corallo F, Marra A, Bramanti P, Calabrò RS. Effect of cranioplasty on functional and neuro -psychological recovery after severe acquired brain injury: fact or fake? Considerations on a single case. Funct Neurol. 2014 Oct-Dec;29(4):273-5. PMID: 25764258; PMCID: PMC4370441.

87: Hao T, Zhu J, Hu W, Zhang H, Gao Z, Wen X, Zhou Z, Lu G, Liu J, Li W. [Application of damage control surgery strategy in treatment of burn-trauma combined injury]. Zhongguo Xiu Fu Chong Jian Wai Ke Za Zhi. 2010 Jun;24(6):661-4. Chinese. PMID: 20632494.

88: Chichevatov D, Gorshenev A, Sinev E. Preventive diaphragm plasty after pneumonectomy on account of lung cancer. Asian Cardiovasc Thorac Ann. 2006 Aug;14(4):265-72. doi: 10.1177/021849230601400401. PMID: 16868097.

89: Moon DB, Lee SG, Ahn CS, Kim KH, Hwang S, Ha TY, Song GW, Jung DH, Ryu JH, Kim KW, Choi NK, Ko G, Sung K, Lee S. Technical modification of reno-portal anastomosis in living donor liver

transplantation for patients with obliterated portal vein and large spontaneous splenorenal shunts. Hepatogastroenterology. 2008 Nov-Dec;55(88):2193-9. PMID: 19260504.

90: Fiorella D, Albuquerque FC, Deshmukh VR, Woo HH, Rasmussen PA, Masaryk TJ, McDougall CG. Endovascular reconstruction with the Neuroform stent as monotherapy for the treatment of uncoilable intradural pseudoaneurysms. Neurosurgery. 2006 Aug;59(2):291-300; discussion 291-300. doi: 10.1227/01.NEU.0000223650.11954.6C. PMID: 16823325.

91: Goldberg RA, Goldey SH, Duckwiler G, Vinuela F. Management of cavernous sinus-dural fistulas. Indications and techniques for primary embolization via the superior ophthalmic vein. Arch Ophthalmol. 1996 Jun;114(6):707-14. doi: 10.1001/archopht.1996.01100130699011. PMID: 8639083.

92: Bojanowski WM, Spetzler RF, Carter LP. Reconstruction of the MCA bifurcation after excision of a giant aneurysm. Technical note. J Neurosurg. 1988 Jun;68(6):974-7. doi: 10.3171/jns.1988.68.6.0974. PMID: 3373294.

93: Xie T, Liu Y, Han T, Zhu S, Zang M, Chen B, Li S. [Flap design and preliminary clinical experience of the lower trapezius musculocutaneous flap carrying a portion of the latissimus dorsi muscle]. Zhongguo Xiu Fu Chong Jian Wai Ke Za Zhi. 2021 Mar 15;35(3):349-355. Chinese. doi: 10.7507/1002-1892.202009114. PMID: 33719245; PMCID: PMC8171762.

94: Nishihara T, Teraoka A, Morita A, Ueki K, Takai K, Kirino T. A transparent sheath for endoscopic surgery and its application in surgical evacuation of spontaneous intracerebral hematomas. Technical note. J Neurosurg. 2000 Jun;92(6):1053-5. doi: 10.3171/jns.2000.92.6.1053. PMID: 10839271.

95: Geller E, Yoon MS, Loiselle J, Crisci KL, Bierbrauer KS. Head injuries in children from plastic hairbeads. Pediatr Radiol. 1997 Oct;27(10):790-3. doi: 10.1007/s002470050234. PMID: 9323242.

96: Osada SI, Kawana S, Saeki H. Non-asthmatic and HCV-seropositive eosinophilic granulomatosis with polyangiitis complicated by multiple intracerebral haemorrhages: a case study. Eur J Dermatol.

2019 Feb 1;29(1):85-87. doi: 10.1684/ejd.2018.3432. PMID: 30499447.

97: Hilbert-Carius P, Heiser A, Wrigge H, Hölbing P, Schröter P, Kobbe P, Großstück A. The Use of Resuscitative Endovascular Balloon Occlusion of the Aorta in a Case of Suspected Septic Distributive Shock: A Case Report. J Am Coll Emerg Physicians Open. 2025 Mar 14;6(3):100088. doi: 10.1016/j.acepjo.2025.100088. PMID: 40171321; PMCID: PMC11957487.

98: Nagayama K, Yoshikawa G, Somekawa K, Kohno M, Segawa H, Sano K, Shiokawa Y, Saito I. [Cranioplasty using the patient's autogenous bone preserved by freezing--an examination of post-operative infection rates]. No Shinkei Geka. 2002 Feb;30(2):165-9. Japanese. PMID: 11857940.

99: Corallo F, Calabro RS, Leo A, Bramanti P. Can cranioplasty be effective in improving cognitive and motor function in patients with chronic disorders of consciousness? A case report. Turk Neurosurg. 2015;25(1):193-6. doi: 10.5137/1019-5149.JTN.10618-14.2. PMID: 25640571.

100: Başkaya MK, Uluç K. Application of a new fenestrated clip (Yaşargil T-bar clip) for the treatment of fusiform M1 aneurysm: case illustration and technical report. Neurosurgery. 2012 Jun;70(2 Suppl Operative):339-42. doi: 10.1227/NEU.0b013e3182330ef7. PMID: 21869719.

101: Katamnez bol'nykh, operirovannykh v ostroĭ stadii razryva arterial'nykh anevrizm [A follow-up of patients operated on for acute arterial aneurysmal rupture]. Zh Vopr Neirokhir Im N N Burdenko. 2004 Jul-Sep;(3):8-13; discussion 13. Russian. PMID: 15490632.

102: Beekmans SJ, Don Griot JP, Mulder JW. Split rib cranioplasty for aplasia cutis congenita and traumatic skull defects: more than 30 years of follow-up. J Craniofac Surg. 2007 May;18(3):594-7. doi: 10.1097/scs.0b013e3180576f44. PMID: 17538324.

103: Menger RP, Valerio IL. Neurological Manifestations of COVID-19 Within the Intensive Care Unit During a Military Deployment for the Early Pandemic Surge in New York City. Cureus. 2021 Mar 12;13(3):e13858. doi: 10.7759/cureus.13858. PMID: 33859908; PMCID: PMC8038911.

104: Takanashi Y, Shinonaga M, Manaka H. Penetrating brain injury with nasal entry by a plastic stick. Case report. J Neurosurg Sci. 2002 Mar;46(1):25-7; discussion 27. PMID: 12118220.

105: Lapras C, Goutelle A. Le traitement des anévrysmes intra-craniens par enrobage plastique [Management of intracranial aneurysms using plastic coating]. Neurochirurgie. 1969 Mar-Apr;15(2):107-12. French. PMID: 5802233.

106: Martello JY, Vasconez HC. Supraorbital roof fractures: a formidable entity with which to contend. Ann Plast Surg. 1997 Mar;38(3):223-7. PMID: 9088458.

107: Ozbek MR, Kutlu N, Khouri RK, Gültan SM, Yormuk E. Prefabricated microvascular shoulder free flap for forehead reconstruction. Handchir Mikrochir Plast Chir. 1993 May;25(3):139-43. PMID: 8330776.

108: Yoshioka N, Haraoka G, Muraoka M, Tominaga S. Single stage reconstruction of scalp and skull using free muscle flap and titanium mesh in patients with epidural infection. J Craniomaxillofac Surg. 1996 Apr;24(2):118-21. doi: 10.1016/s1010-5182(96)80023-0. PMID: 8773894.

109: Lee JW, Hsueh YY, Lee JS. Composite skull and dura defect reconstruction using combined latissimus dorsi musculocutaneous and serratus anterior muscle-rib free flap coupled with vascularized galea transfer: a case report. Microsurgery. 2010 Nov;30(8):632-5. doi: 10.1002/micr.20808. Epub 2010 Aug 23. PMID: 20734432.

110: Salzano A, Nocera V, De Rosa A, Rossi E, Nunziata A, Tuccillo M, Brunese L, Grassi R. Traumi cranio-encefalici da proiettile: correlazione tra Tomografia Computerizzata, clinica, trattamento neurochirurgico e sequele a distanza [Craniocerebral trauma from bullets: the correlation between computed tomography, the clinical picture, neurosurgical treatment and the long-term sequelae]. Radiol Med. 2000 Mar;99(3):156-60. Italian. PMID: 10879162.

111: Watanabe A, Seguchi T, Hongo K. Overdrainage of cerebrospinal fluid caused by detachment of the pressure control cam in a programmable valve after 3-tesla magnetic resonance imaging. J Neurosurg. 2010 Feb;112(2):425-7. doi: 10.3171/2008.3.17688. PMID: 19408984.

112: Khil'ko VA, Golovashchenko NV. Profuznye nosovye krovotecheniia, obuslovlennye travmaticheskim povrezhdeniem subklinoidnoĭ chasti vnutrenneĭ sonnoĭ arterii [Profuse nasal hemorrhages caused by traumatic damage to the subclinoid portion of the internal carotid artery]. Zh Vopr Neirokhir Im N N Burdenko. 1980 Mar-Apr;(2):9-13. Russian. PMID: 7386099.

113: Benson CM, Joswig H, Evans HB, Steven DA. Case Report: Free Latissimus Dorsi Flap in Combination With Subdural Space Reduction for the Prevention of Recurrent Hemorrhage Following Hemispherectomy. Oper Neurosurg. 2018 Jun 1;14(6):E63-E65. doi: 10.1093/ons/opx159. PMID: 28973550.

114: Dong J, Wang S, Bie P, Wang H, Yang Z, He Y, Li Z, Cai J. [Experience of vascular and bile duct reconstruction in 40 cases of orthotopic liver transplantation]. Zhonghua Gan Zang Bing Za Zhi. 2002 Feb;10(1):10-3. Chinese. PMID: 11856490.

115: Li Y, Horiuchi T, Nakagawa F, Hongo K. Vertebral artery dissecting aneurysm treated by proximal occlusion and posterior inferior cerebellar artery reconstruction with fenestrated clips. Case report. Neurol Med Chir (Tokyo). 2010;50(8):655-8. doi: 10.2176/nmc.50.655. PMID: 20805648.

116: Wang C, Wang X. Cerebral Hemorrhage after Cosmetic Facial Injection. Plast Reconstr Surg Glob Open. 2019 Sep 10;7(9):e2397. doi: 10.1097/GOX.0000000000002397. PMID: 31942378; PMCID: PMC6908375.

117: Thimukonda Jegadeesan J, Baldia M, Basu B. Next-generation personalized cranioplasty treatment. Acta Biomater. 2022 Dec;154:63-82. doi: 10.1016/j.actbio.2022.10.030. Epub 2022 Oct 19. PMID: 36272686.

118: Global Burden of Cardiovascular Diseases and Risks 2023 Collaborators. Global, Regional, and National Burden of Cardiovascular Diseases and Risk Factors in 204 Countries and Territories, 1990-2023. J Am Coll Cardiol. 2025 Dec 2;86(22):2167-2243. doi: 10.1016/j.jacc.2025.08.015. Epub 2025 Sep 24. PMID: 40990886.

119: Bader ER, Allam MM, Harris TG, Suchdev N, Loke YK, Barlas R. Thrombolysis for aneurysmal subarachnoid haemorrhage. Cochrane Database Syst Rev. 2025 Jan 17;1(1):CD013748. doi: 10.1002/14651858.CD013748.pub2. PMID: 39822092; PMCID: PMC11740285.

120: Wang HC, Yu N, Wang X, Dong R, Long X, Feng X, Li J, Wu WTL. Cerebral Embolism as a Result of Facial Filler Injections: A Literature Review. Aesthet Surg J. 2022 Feb 15;42(3):NP162-NP175. doi: 10.1093/asj/sjab193. PMID: 33856432; PMCID: PMC8844978.

121: Khanna O, Baldassari MP, Al Saiegh F, Mouchtouris N, Ghosh R, Theofanis TN, Evans JJ, Tjoumakaris S, Rosenwasser RH, Jabbour PM, Gooch MR. Ultrasound-Guided Ventricular Puncture During Cranioplasty. World Neurosurg. 2021 Feb;146:e779-e785. doi: 10.1016/j.wneu.2020.11.021. Epub 2020 Nov 10. PMID: 33181379.

122: Chen Y, Wu G, Zhang W, Zou Z, Huang L, Shi H, Wang Q, Chen W, Luo Z, Zhao Z, Wu L, Li Z, Peng J, Chen Y, Zhang JH. Decoding spontaneous intracerebral hemorrhage: mechanistic breakthroughs and disruptive revolution in pharmacological treatment. Exp Mol Med. 2026 May;58(5):1394-1408. doi: 10.1038/s12276-026-01733-z. Epub 2026 May 28. PMID: 42209696; PMCID: PMC13234291.

123: Chen C, Huang CY, Wang HJ, Chen CI, Lin HW. Stroke after burn: population data analysis. Burns. 2014 Mar;40(2):230-4. doi: 10.1016/j.burns.2013.10.002. Epub 2013 Nov 23. PMID: 24280523.

124: El Mohamad AR, Khan MM, Omari RY, Strandvik G. Massive traumatic subarachnoid hemorrhage mimicking aneurysmal subarachnoid hemorrhage. Trauma Case Rep. 2023 Oct 19;48:100959. doi: 10.1016/j.tcr.2023.100959. PMID: 37915535; PMCID: PMC10616423.

125: Wehman JC, Hanel RA, Levy EI, Hopkins LN. Giant cerebral aneurysms: endovascular challenges. Neurosurgery. 2006 Nov;59(5 Suppl 3):S125-38; discussion S3-13. doi: 10.1227/01.NEU.0000237330.11482.90. PMID: 17053595.

126: Hsieh CH, Liu HT, Hsu SY, Hsieh HY, Chen YC. Motorcycle-related hospitalizations of the elderly. Biomed J. 2017 Apr;40(2):121-128. doi: 10.1016/j.bj.2016.10.006. Epub 2017 May 8. PMID: 28521903; PMCID: PMC6138602.

127: Chen Y, Li G, Chen X, Wang D, Fang W, Kang D, Ding C. Non-cerebral vasospasm factors and cerebral vasospasm predict delayed cerebral ischemia after aneurysmal subarachnoid hemorrhage. Chin Med J (Engl). 2021 Dec 13;135(2):222-224. doi: 10.1097/CM9.0000000000001844. PMID: 34908005; PMCID: PMC8769126.

128: Rennert RC, Atai NA, Nguyen VN, Abedi A, Sternbach S, Chu J, Carey JN, Russin JJ. Three-Vessel Anastomosis for Direct Multiterritory Cerebral Revascularization: Case Series. Oper Neurosurg. 2024 Apr 1;26(4):423-432. doi: 10.1227/ons.0000000000001013. Epub 2023 Dec 12. PMID: 38084991.

129: Tos SM, Osama M, Mantziaris G, Hajikarimloo B, Adeeb N, Kandregula S, Dmytriw AA, Salim HA, Musmar B, Naamani KE, Ogilvy C, Kondziolka D, Abdelsalam A, Kumbhare D, Gummadi S, Ataoglu C, Erginoglu U, Essibayi MA, Keles A, Muram S, Sconzo D, Riina H, Rezai A, Pöppe J, Sen RD, Alwakaa O, Griessenauer CJ, Jabbour P, Tjoumakaris SI, Burkhardt JK, Starke RM, Baskaya M, Sekhar LN, Levitt MR, Altschul DJ, Haranhalli N, McAvoy M, Aslan A, Abushehab A, Swaid C, Abla A, Stapleton C, Koch M, Srinivasan VM, Chen PR, Blackburn S, Dannenbaum MJ, Choudhri O, Pukenas B, Orbach D, Smith E, Möhlenbruch M, Alaraj A, Aziz-Sultan A, Patel AB, Cuellar HH, Lawton M, Morcos J, Guthikonda B, Sheehan J. Spetzler-Martin grade I and II cerebral arteriovenous malformations: a propensity-score matched analysis of resection and stereotactic radiosurgery in adult patients. Neurosurg Rev. 2025 Feb 28;48(1):276. doi: 10.1007/s10143-025-03431-2. PMID: 40016553; PMCID: PMC11868255.

130: Sajjad S, Hewera M, Rana M, Gliem M, Fischer I, Khan D. Neutrophils extracellular traps myeloperoxidase and elastase predict cerebral vasospasm after aneurysmal subarachnoid hemorrhage. Heliyon. 2024 Nov 20;10(23):e40562. doi: 10.1016/j.heliyon.2024.e40562. PMID: 39654759; PMCID:

PMC11625263.

131: Ohuchi H, Takei K, Muneuchi J, Kasahara S, Ishikawa Y, Muraji S, Tsukada M, Nii M, Ono S, Takamuro M, Saiki H, Fujino M, Kuraishi K, Lin L, Miyazaki A, Sakamoto I, Masutani S, Hayabuchi Y, Ohashi H, Yasuda K, Seki M, Mori A, Nakai M. Unplanned Hospitalization Due to All-Cause Morbidity and Its Real-World Management Practices in Patients With Fontan Circulation. J Am Heart Assoc. 2025 Dec 16;14(24):e044374. doi: 10.1161/JAHA.125.044374. Epub 2025 Dec 10. PMID: 41368820; PMCID: PMC12826895.

132: Albuquerque Sousa LH, Maranha Gatto LA, Demartini Junior Z, Koppe GL. Scalp Cirsoid Aneurysm: An Updated Systematic Literature Review and an Illustrative Case Report. World Neurosurg. 2018 Nov;119:416-427. doi: 10.1016/j.wneu.2018.08.098. Epub 2018 Aug 24. PMID: 30149169.

133: Zhang J, Liu X, Zhou J, Zhang Z, Fu M, Guo Y, Li G. Seizures Following Cranioplasty: Risk Factors and Prevention Exploration. J Craniofac Surg. 2019 Mar/Apr;30(2):e170-e175. doi: 10.1097/SCS.0000000000005116. PMID: 30653038.

134: Nagai A, Kimura N, Uchida H, Yokosawa M, Degawa K, Sugawara T, Tominaga T. Ultra-High-Molecular-Weight Polyethylene Merlon Shape: Novel Fixation of Artificial Bone for Cranioplasty. Oper Neurosurg. 2023 Apr 1;24(4):404-409. doi: 10.1227/ons.0000000000000565. Epub 2022 Dec 16. PMID: 36701690; PMCID: PMC9974084.

135: Staffenberg DA, Goodrich JT. Separation of craniopagus conjoined twins with a staged approach. J Craniofac Surg. 2012 Nov;23(7 Suppl 1):2004-10. doi: 10.1097/SCS.0b013e318262d3f7. PMID: 23154370.

136: Zhang S, Zhuang J, Wu W, Huang L, Tian M, Guo L, Sun M, Hu J, Liu N. What Factors Can Affect the Occurrence of Vertigo in Patients After Craniofacial Surgery in China? J Craniofac Surg. 2024 Jun 1;35(4):e408-e411. doi: 10.1097/SCS.0000000000010067. Epub 2024 Mar 27. PMID: 38534183.

137: Chim H, Arcelona C, Gosain AK. Scalp Closure in Midline Cutis Aplasia-An Absolute Indication for Preoperative Imaging. J Craniofac Surg. 2024 Jun 1;35(4):e345-e347. doi: 10.1097/SCS.0000000000010062. Epub 2024 Feb 23. PMID: 38393191.

138: Greene AK, Burrows PE, Smith L, Mulliken JB. Periorbital lymphatic malformation: clinical course and management in 42 patients. Plast Reconstr Surg. 2005 Jan;115(1):22-30. PMID: 15622227.

139: Sadiq KO, Desai S, Miller S, Abualnadi YD, Khalil ZM, Khan Z, Amjadi N, Ravindra VM, Tekle W, Georgiadis AL, Hassan AE. Epidural anesthesia causes outbreak of mycotic aneurysms: complications of <i>Fusarium solani</i> meningitis. J Neurointerv Surg. 2024 Feb 28:jnis-2023-021300. doi: 10.1136/jnis-2023-021300. Epub ahead of print. PMID: 38418228.

140: Tos SM, Hajikarimloo B, Osama M, Mantziaris G, Adeeb N, Kandregula S, Salim HA, Musmar B, Ogilvy C, Kondziolka D, Dmytriw AA, El Naamani K, Abdelsalam A, Kumbhare D, Gummadi S, Ataoglu C, Essibayi MA, Erginoglu U, Keles A, Muram S, Sconzo D, Riina H, Rezai A, Pöppe J, Sen RD, Alwakaa O, Griessenauer CJ, Jabbour P, Tjoumakaris SI, Burkhardt JK, Starke RM, Baskaya MK, Sekhar LN, Levitt MR, Altschul DJ, Haranhalli N, McAvoy M, Abushehab A, Aslan A, Swaid C, Abla A, Stapleton C, Koch M, Srinivasan VM, Chen PR, Blackburn S, Cochran J, Choudhri O, Pukenas B, Orbach D, Smith E, Möhlenbruch M, Alaraj A, Aziz-Sultan A, Dlouhy K, El Ahmadieh T, Patel AB, Savardekar A, Cuellar HH, Lawton M, Guthikonda B, Morcos J, Sheehan J. A comparative analysis of microsurgical resection versus stereotactic radiosurgery for Spetzler-Martin grade III arteriovenous malformations: A multicenter propensity score matched study. Clin Neurol Neurosurg. 2025 Feb;249:108669. doi: 10.1016/j.clineuro.2024.108669. Epub 2024 Dec 3. PMID: 39642799.

141: Zhang X, Pan B, Ye Z, Li Z, Mo F, Wang X. Massive Brain Swelling after Cranioplasty: A Case Report. J Neurol Surg A Cent Eur Neurosurg. 2019 Nov;80(6):498-502. doi: 10.1055/s-0039-1688726. Epub 2019 May 10. PMID: 31075809.

142: Morton RP, Abecassis IJ, Hanson JF, Barber JK, Chen M, Kelly CM, Nerva JD, Emerson SN, Ene CI, Levitt MR, Chowdhary MM, Ko AL, Chesnut RM. Timing of cranioplasty: a 10.75-year single-center analysis of 754 patients. J Neurosurg. 2018 Jun;128(6):1648-1652. doi:

10.3171/2016.11.JNS161917. Epub 2017 Aug 11. PMID: 28799868.

143: Chalela JA, Katzan I, Liebeskind DS, Rasmussen P, Zaidat O, Suarez JI, Chiu D, Klucznick RP, Jauch E, Cucchiara BL, Saver J, Kasner SE. Safety of intra-arterial thrombolysis in the postoperative period. Stroke. 2001 Jun;32(6):1365-9. doi: 10.1161/01.str.32.6.1365. PMID: 11387500.

144: van Rooij WJ, Sluzewski M. Endovascular treatment of large and giant aneurysms. AJNR Am J Neuroradiol. 2009 Jan;30(1):12-8. doi: 10.3174/ajnr.A1267. Epub 2008 Aug 21. PMID: 18719032; PMCID: PMC7051694.

145: Ma J, Huang X, Hu Y, Xu B, Jin C. Clinical Value of Serum Secretoneurin Levels in Prediction of Delayed Cerebral Ischemia and Prognostic Analysis of Aneurysmal Subarachnoid Hemorrhage: A Prospective Cohort Study. Int J Gen Med. 2024 Aug 16;17:3555-3573. doi: 10.2147/IJGM.S469287. PMID: 39165486; PMCID: PMC11334926.

146: Koebbe CJ, Veznedaroglu E, Jabbour P, Rosenwasser RH. Endovascular management of intracranial aneurysms: current experience and future advances. Neurosurgery. 2006 Nov;59(5 Suppl 3):S93-102; discussion S3-13. doi: 10.1227/01.NEU.0000237512.10529.58. PMID: 17053622.

147: Gao S, Liu XZ, Wu LY, Peng Z, Chen XX, Wang H, Lu Y, Zhuang Z, Tan Q, Hang CH, Li W. Long-Term Elevated Siglec-10 in Cerebral Spinal Fluid Heralds Better Prognosis for Patients with Aneurysmal Subarachnoid Hemorrhage. Dis Markers. 2022 Sep 21;2022:5382100. doi: 10.1155/2022/5382100. PMID: 36188429; PMCID: PMC9519311.

148: Ryu JY, Chang YJ, Lee JS, Choi KY, Yang JD, Lee SJ, Lee J, Huh S, Kim JY, Chung HY. Extracranial Vascular Malformations Increase Cardiovascular Disease Risk: A Nationwide Population-Based Cohort Study. Plast Reconstr Surg. 2024 Nov 1;154(5):1047e-1058e. doi: 10.1097/PRS.0000000000011297. Epub 2024 Jan 17. PMID: 38232222; PMCID: PMC11512613.

149: Roth C, Salehi M, Deinsberger W, Kaestner S, Engel H. Conservative versusoperative treatment

in supratentorial intracerebral hemorrhage - A survey among neurosurgeons and neurologists in Germany. Clin Neurol Neurosurg. 2019 Nov;186:105502. doi: 10.1016/j.clineuro.2019.105502. Epub 2019 Aug 27. PMID: 31494462.

150: Jhou HJ, Lee CH, Tsai YC, Chen PH, Yang LY. Is Thrombectomy Worth It for Isolated Posterior Cerebral Artery Occlusion? Meta-Analysis and Trial Sequential Analysis. Stroke Vasc Interv Neurol. 2024 Feb 7;4(2):e001084. doi: 10.1161/SVIN.123.001084. PMID: 41583603; PMCID: PMC12778484.

151: Mendenhall SK, Shapiro SA, Cohen-Gadol AA, Sahlein DH. Endovascular Retreatment of Previously Ruptured Coiled Cerebral Aneurysm Remnants Significantly Reduces Rebleed Rate. World Neurosurg. 2021 Mar;147:e382-e387. doi: 10.1016/j.wneu.2020.12.063. Epub 2020 Dec 19. PMID: 33352305.

152: Carbonaro R, Ghiringhelli G, Nataloni A, Amendola F, Catapano S, Vaienti L, Umana GE, Fricia M, Zingaretti N, Zanotti B. Long-Term Series of Custom-Bone Hydroxyapatite Cranioplasty: Outcomes and Survival at 15 Years. J Craniofac Surg. 2025 Jun 1;36(4):1263-1266. doi: 10.1097/SCS.0000000000011061. Epub 2025 Jan 3. PMID: 39953654.

153: Cimbanassi S, O'Toole R, Maegele M, Henry S, Scalea TM, Bove F, Mezzadri U, Capitani D, Sala F, Kanakaris N, Coccolini F, Ansaloni L, Sgardello S, Bindi F, Renzi F, Sammartano F, Masse A, Rampoldi A, Puoti M, Berlusconi M, Moretti B, Rueger J, Arnez Z, Del Bene M, Chieregato A, Menarini M, Gordini G, De Blasio E, Cudoni S, Dionigi P, Fabbri A, Scandroglio I, Chiara O. Orthopedic injuries in patients with multiple injuries: Results of the 11th trauma update international consensus conference Milan, December 11, 2017. J Trauma Acute Care Surg. 2020 Feb;88(2):e53-e76. doi: 10.1097/TA.0000000000002407. PMID: 32150031.

154: Song TW, Lee SK, Kim TS, Joo SP. Pericranial Flap Anterior Skull Base Reconstruction With Gelfoam Intradural Compression. J Craniofac Surg. 2019 Jun;30(4):1280-1283. doi: 10.1097/SCS.0000000000005364. PMID: 30921076.

155: Murakami D, Kuga D, Miyamoto Y, Komune N, Mukae N, Saito Y, Iihara K, Nakagawa T. A Pedicled Posterior Septal-Nasal Floor Flap and a Novel Rescue Flap for Skull Base Reconstruction. World Neurosurg. 2021 Jun;150:197-204.e1. doi: 10.1016/j.wneu.2021.02.138. Epub 2021 Mar 6. PMID: 33689848.

156: Li H, Zhang S. Massive pneumocephalus after mechanical ventilation in patient with intracerebral hemorrhage. Neurol Sci. 2023 Mar;44(3):1121-1123. doi: 10.1007/s10072-022-06474-1. Epub 2022 Nov 8. PMID: 36344768.

157: Christianson D, Seaman SC, Ray E, Li L, Zanaty M, Lemoine P, Wilson G, Grimm D, Park BJ, Gold C, Andrews B, Grady S, Dlouhy K, Howard MA 3rd. The Adjustable Cranial Plate: A Novel Implant Designed to Eliminate the Need for Cranioplasty Surgery Following a Hemicraniectomy Operation. World Neurosurg. 2023 May;173:e306-e320. doi: 10.1016/j.wneu.2023.02.045. Epub 2023 Feb 15. PMID: 36804433.

158: Makino K, Tsutsumi S, Takaki Y, Nonaka S, Okura H, Ishii H. Infantile acute subdural hemorrhage probably caused by injury to the diploic channels. Radiol Case Rep. 2021 Aug 5;16(10):2981-2983. doi: 10.1016/j.radcr.2021.07.023. PMID: 34401037; PMCID: PMC8353404.

159: Lan S, Zhou L, Wang Y, Fang L, Yang L, Zheng S, Zhou X, Tang B, Duan J, Wu X, Yang C, Hong T. miRNA Profiling of Circulating Small Extracellular Vesicles From Subarachnoid Hemorrhage Rats Using Next-Generation Sequencing. Front Cell Neurosci. 2020 Aug 13;14:242. doi: 10.3389/fncel.2020.00242. PMID: 32903819; PMCID: PMC7439219.

160: Kumar R, Deleyiannis FW, Wilkinson C, O'Neill BR. Neurosurgical sequelae of domestic dog attacks in children. J Neurosurg Pediatr. 2017 Jan;19(1):24-31. doi: 10.3171/2016.7.PEDS1646. Epub 2016 Oct 21. PMID: 27767903.

161: Herrera SR, Shin JH, Chan M, Kouloumberis P, Goellner E, Slavin KV. Use of transparent plastic tubular retractor in surgery for deep brain lesions: a case series. Surg Technol Int. 2010 Apr;19:47-50. PMID: 20437344.

162: DECAMP PT, OCHSNER A Jr. Reconstructive surgical procedures for cerebrovascular insufficiency. J La State Med Soc. 1959 Mar;111(3):85-91. PMID: 13641875.

163: Dorobisz AT, Rybak Z, Skóra J, Pupka A, Patrzałek D, Stepiński P, Korta K, Barć P. Iatrogenic injuries of the carotid arteries. Vasa. 2005 Aug;34(3):192-4. doi: 10.1024/0301-1526.34.3.192. PMID: 16184839.

164: Shin KJ, Lee DG, Park HM, Choi MY, Bae JH, Lee ET. The merits of mannitol in the repair of orbital blowout fracture. Arch Plast Surg. 2013 Nov;40(6):721-7. doi: 10.5999/aps.2013.40.6.721. Epub 2013 Nov 8. PMID: 24286045; PMCID: PMC3840179.

165: Zhao WY, Zhao KJ, Huang QH, Xu Y, Hong B, Liu JM. Single-stage endovascular treatment of subarachnoid hemorrhage related to bilateral vertebral artery dissecting aneurysms. Interv Neuroradiol. 2016 Apr;22(2):138-42. doi: 10.1177/1591019915617325. Epub 2015 Dec 18. PMID: 26686384; PMCID: PMC4984341.

166: Charcos IB, Wong TW, Larsen BR, Azurdia AR, Gridley DG, Vail SJ, Hollingworth AK, Lettieri SC, Feiz-Erfan I. Location of Traumatic Cranial Epidural Hematoma Correlates with the Source of Hemorrhage: A 12-Year Surgical Review. World Neurosurg. 2021 Aug;152:e138-e143. doi: 10.1016/j.wneu.2021.05.052. Epub 2021 May 24. PMID: 34033954.

167: Pei H, Peng Q, Guo S, Gu Y, Sun T, Xu D, Jiang Y, Xie J, Zhang L, Zhu Z. MiR-367 alleviates inflammatory injury of microglia by promoting M2 polarization via targeting CEBPA. In Vitro Cell Dev Biol Anim. 2020 Dec;56(10):878-887. doi: 10.1007/s11626-020-00519-5. Epub 2020 Nov 4. PMID: 33150481; PMCID: PMC7723938.

168: Kim EG, Eom TK, Kang SJ. Severe visual loss and cerebral infarction after injection of hyaluronic acid gel. J Craniofac Surg. 2014;25(2):684-6. doi: 10.1097/SCS.0000000000000537. PMID: 24621723.

169: Alomari MH, Kozakewich HPW, Kerr CL, Uller W, Davis SL, Chaudry G, Liang MG, Orbach DB, Mulliken JB, Greene AK, Afshar S, Fishman SJ, Taghinia AH, Al-Ibraheemi A, Alomari AI. Congenital Disseminated Pyogenic Granuloma: Characterization of an Aggressive Multisystemic Disorder. J Pediatr. 2020 Nov;226:157-166. doi: 10.1016/j.jpeds.2020.06.079. Epub 2020 Jul 2. PMID: 32622671.

170: Gu H, Zhang E, Li B, Shi L, He M, Xu S, Shi G. [Clinical analysis of resection and dura skull base reconstruction of cranionasal communication tumor in 31 cases]. Lin Chuang Er Bi Yan Hou Tou Jing Wai Ke Za Zhi. 2024 Jun;38(6):496-502. Chinese. doi: 10.13201/j.issn.2096-7993.2024.06.008. PMID: 38858114; PMCID: PMC11480574.

171: Zhou JH, Wang JL, Yang D, Wu YX, Zhang W, Qin HZ, Wang C, Li J. Characteristics and Clinical Significance of Skull Defect Restoration in Young Patients: A Single-center Report and Literature Review. J Craniofac Surg. 2025 Jul-Aug 01;36(5):1655-1660. doi: 10.1097/SCS.0000000000010851. Epub 2024 Nov 11. PMID: 39527727.

172: Ravina K, Bakhsheshian J, Carey JN, Russin JJ. External Carotid Artery-to-Middle Cerebral Artery Bypass Using a Saphenous Vein Graft With 3-Vessel Anastomosis for the Treatment of a Large, Ruptured Middle Cerebral Artery Aneurysm: 2-Dimensional Operative Video. Oper Neurosurg. 2021 Apr 15;20(5):E359-E360. doi: 10.1093/ons/opaa426. PMID: 33442742.

173: Hekmatpanah J. Cerebral microvessel perfusion and pathologic alteration of the brain during drowsiness and coma caused by brain tumor: a laboratory study on rats. Surg Neurol. 2007 Jun;67(6):564-71; discussion 571. doi: 10.1016/j.surneu.2006.09.030. Epub 2007 Mar 26. PMID: 17368521; PMCID: PMC2042104.

174: di Rocco F, Gleizal A, Lohkamp L, Szathmari A, Paulus C, Mottolese C. Control of metopic emissary veins in trigonocephaly surgery. Technical note. Childs Nerv Syst. 2018 Dec;34(12):2481-2484. doi: 10.1007/s00381-018-3928-1. Epub 2018 Jul 27. PMID: 30054806.

175: Wang S, Cui Y, Xu J, Gao H. miR-140-5p Attenuates Neuroinflammation and Brain Injury in Rats

Following Intracerebral Hemorrhage by Targeting TLR4. Inflammation. 2019 Oct;42(5):1869-1877. doi: 10.1007/s10753-019-01049-3. PMID: 31376096.

176: Fountain DM, Henry J, Honeyman S, O'Connor P, Sekhon P, Piper RJ, Edlmann E, Martin M, Whiting G, Turner C, Mee H, Joannides AJ, Kolias AG, Hutchinson PJ; UK Cranial Reconstruction Registry (UKCRR) Collaborative; British Neurosurgical Trainee Research Collaborative (BNTRC). First Report of a Multicenter Prospective Registry of Cranioplasty in the United Kingdom and Ireland. Neurosurgery. 2021 Aug 16;89(3):518-526. doi: 10.1093/neuros/nyab220. PMID: 34192745.

177: Kotlus BS, Lo MW. Subarachnoid hemorrhage and vasospastic stroke after self-enucleation. Ophthalmic Plast Reconstr Surg. 2007 Sep-Oct;23(5):425-7. doi: 10.1097/IOP.0b013e31814e0527. PMID: 17882004.

178: Zhang J, Peng F, Liu Z, Luan J, Liu X, Fei C, Heng X. Cranioplasty with autogenous bone flaps cryopreserved in povidone iodine: a long-term follow-up study. J Neurosurg. 2017 Dec;127(6):1449-1456. doi: 10.3171/2016.8.JNS16204. Epub 2017 Feb 10. PMID: 28186447.

179: Zi L, Zhou W, Xu J, Li J, Li N, Xu J, You C, Wang C, Tian M. Rosuvastatin Nanomicelles Target Neuroinflammation and Improve Neurological Deficit in a Mouse Model of Intracerebral Hemorrhage. Int J Nanomedicine. 2021 Apr 20;16:2933-2947. doi: 10.2147/IJN.S294916. PMID: 33907400; PMCID: PMC8068519.

180: Lewis KM, Sweet J, Wilson ST, Rousselle S, Gulle H, Baumgartner B. Safety and Efficacy of a Novel, Self-Adhering Dural Substitute in a Canine Supratentorial Durotomy Model. Neurosurgery. 2018 Mar 1;82(3):397-406. doi: 10.1093/neuros/nyx216. PMID: 28575349; PMCID: PMC6018778.

181: Konczalla J, Platz J, Fichtlscherer S, Mutlak H, Strouhal U, Seifert V. Rapid ventricular pacing for clip reconstruction of complex unruptured intracranial aneurysms: results of an interdisciplinary prospective trial. J Neurosurg. 2018 Jun;128(6):1741-1752. doi: 10.3171/2016.11.JNS161420. Epub 2017

Aug 18. PMID: 28820303.

182: Marano AA, Hoppe IC, Halsey JN, Kordahi AM, Granick MS, Lee ES. Patterns of Intracranial Hemorrhage in Pediatric Patients with Facial Fractures. Craniomaxillofac Trauma Reconstr. 2016 Mar;9(1):35-9. doi: 10.1055/s-0035-1558453. Epub 2015 Jul 24. PMID: 26889346; PMCID: PMC4755725.

183: Zhang L, Sun XG, Yu CT, Chang Q, Qian XY. Intercostal Artery Reconstruction: The Simple and Effective Technique on Spinal Cord Protection during Thoracoabdominal Aortic Replacement. Ann Vasc Surg. 2016 Jul;34:62-7. doi: 10.1016/j.avsg.2015.12.030. Epub 2016 May 10. PMID: 27177704.

184: Christiansen ME, Kumar G, Mahabir RC, Helmers RA, Bendok BR, O'Carroll CB. Intravenous Alteplase for Acute Stroke and Pulmonary Embolism in a Patient With Recent Abdominoplasty. Neurologist. 2017 Jul;22(4):150-152. doi: 10.1097/NRL.0000000000000139. PMID: 28644260.

185: Rachatte MG, Pahari S, Pande A, Mohanty P, Vasudevan MC, Rokaya P, Raut U. Factors Predicting Poor Outcomes Following Cranioplasty: A Single Center Analytical Study. World Neurosurg. 2025 Jun;198:123957. doi: 10.1016/j.wneu.2025.123957. Epub 2025 Apr 10. PMID: 40204214.

186: Oakley GM, Christensen JM, Winder M, Jonker BP, Davidson A, Steel T, Teo C, Harvey RJ. Collagen matrix as an inlay in endoscopic skull base reconstruction. J Laryngol Otol. 2018 Mar;132(3):214-223. doi: 10.1017/S0022215117001499. Epub 2017 Jul 18. PMID: 28716164.

187: Wang Z, Guo S, Wang J, Shen Y, Zhang J, Wu Q. Nrf2/HO-1 mediates the neuroprotective effect of mangiferin on early brain injury after subarachnoid hemorrhage by attenuating mitochondria-related apoptosis and neuroinflammation. Sci Rep. 2017 Sep 19;7(1):11883. doi: 10.1038/s41598-017-12160-6. PMID: 28928429; PMCID: PMC5605716.

188: Hong JW, Chung SW, Ahn SJ, Lee WJ, Lew DH, Kim YO. Analysis of factors involved in brain-death donor processing for face transplantation in Korea: How much time is available from brain death

to transplantation? Arch Plast Surg. 2019 Sep;46(5):405-413. doi: 10.5999/aps.2019.00150. Epub 2019 Aug 30. PMID: 31462025; PMCID: PMC6759447.

189: Hayashi K, Suyama K, Nagata I. Traumatic carotid cavernous fistula complicated with intracerebral hemorrhage: case report. Neurol Med Chir (Tokyo). 2011;51(3):214-6. doi: 10.2176/nmc.51.214. PMID: 21441738.

190: Cellini L, Vaiano A, Tiezzi G, Oliveri G, Di Pietro G, Carangelo BR, Gabriele G, Zerini F, Cascino F, Luglietto D. Complex craniomaxillofacial gunshot wounds A step-by-step multidisciplinary approach. Ann Ital Chir. 2020 Nov 30;91:S2239253X20033125. PMID: 33295298.

191: Yamazaki N, Takenouchi T, Fujimoto M, Ihn H, Uchi H, Inozume T, Kiyohara Y, Uhara H, Nakagawa K, Furukawa H, Wada H, Noguchi K, Shimamoto T, Yokota K. Phase 1b study of pembrolizumab (MK-3475; anti-PD-1 monoclonal antibody) in Japanese patients with advanced melanoma (KEYNOTE-041). Cancer Chemother Pharmacol. 2017 Apr;79(4):651-660. doi: 10.1007/s00280-016-3237-x. Epub 2017 Mar 11. PMID: 28283736; PMCID: PMC5364262.

192: Ishida A, Tamakoshi K, Hamakawa M, Shimada H, Nakashima H, Masuda T, Hida H, Ishida K. Early onset of forced impaired forelimb use causes recovery of forelimb skilled motor function but no effect on gross sensory-motor function after capsular hemorrhage in rats. Behav Brain Res. 2011 Nov 20;225(1):126-34. doi: 10.1016/j.bbr.2011.06.036. Epub 2011 Jul 12. PMID: 21771615.

193: Meyer B, Schaller K, Rohde V, Hassler W. Percutaneous needle trephination. Experience in 200 cases. Acta Neurochir (Wien). 1994;127(3-4):232-5. doi: 10.1007/BF01808772. PMID: 7942209.

194: Zivkovic B, Micovic M, Bascarevic V, Savic A, Rasulic L. Simultaneous bone remodeling and surgical decompression of large Sylvian arachnoid cyst. Neurosurg Rev. 2018 Apr;41(2):683-688. doi: 10.1007/s10143-018-0954-0. Epub 2018 Feb 21. PMID: 29464388.

195: Wang C, Zhang J, Liu Z. Vacuum-assisted closure therapy combined with bi-pectoral muscle flap

for the treatment of deep sternal wound infections. Int Wound J. 2020 Apr;17(2):332-338. doi: 10.1111/iwj.13277. Epub 2019 Dec 1. PMID: 31788960; PMCID: PMC7949189.

196: Kim SW, Han HH, Oh DY, Moon SH, Lee JH, Rhie JW, Ahn ST. Orbital roof reconstruction using porous polyethylene sheet with embedded titanium. J Craniofac Surg. 2012 Nov;23(6):e64-6. doi: 10.1097/SCS.0b013e31825d0b91. PMID: 23172508.

197: Du H, Li Z, Li M, Li L. [CLINICAL RESEARCH AND DISCUSSION OF MODIFIEDCRANIOPLASTY]. Zhongguo Xiu Fu Chong Jian Wai Ke Za Zhi. 2015 Jul;29(7):822-5. Chinese. PMID: 26540973.

198: Yoshioka N. Immediate Cranioplasty for Postcranioplasty Infection in Patients with Ventriculoperitoneal Shunt. World Neurosurg. 2018 Nov;119:311-314. doi: 10.1016/j.wneu.2018.08.076. Epub 2018 Aug 23. PMID: 30144602.

199: Isago T, Nozaki M, Kikuchi Y, Honda T, Nakazawa H. Sinking skin flap syndrome: a case of improved cerebral blood flow after cranioplasty. Ann Plast Surg. 2004 Sep;53(3):288-92. doi: 10.1097/01.sap.0000106433.89983.72. PMID: 15480019.

200: Tung TC, Tseng WS, Chen CT, Lai JP, Chen YR. Acute life-threatening injuries in facial fracture patients: a review of 1,025 patients. J Trauma. 2000 Sep;49(3):420-4. doi: 10.1097/00005373-200009000-00006. PMID: 11003317.

201: Cai B, Yuan P, Fan LN, Ma L, Niu TT, Bai R. Intravenous thrombolysis for acute cerebral embolism during atrial fibrillation ablation: a case report and systematic review with meta-analysis. Front Cardiovasc Med. 2026 Apr 17;13:1801300. doi: 10.3389/fcvm.2026.1801300. PMID: 42079704; PMCID: PMC13133009.

202: Yoshioka N, Tominaga S. Titanium Mesh Implant Exposure Due To Pressure Gradient Fluctuation. World Neurosurg. 2018 Nov;119:e734-e739. doi: 10.1016/j.wneu.2018.07.255. Epub 2018 Aug 6.

PMID: 30092473.

203: Zhang Y, Yi B, Ma J, Zhang L, Zhang H, Yang Y, Dai Y. Quercetin promotes neuronal and behavioral recovery by suppressing inflammatory response and apoptosis in a rat model of intracerebral hemorrhage. Neurochem Res. 2015 Jan;40(1):195-203. doi: 10.1007/s11064-014-1457-1. Epub 2014 Dec 28. PMID: 25543848.

204: Lee HB, Park SW, Kim IK, Kim JH, Kim DY, Hwang KC. Adipose tissue derived stromal vascular fraction as an adjuvant therapy in stroke rehabilitation: Case reports. Medicine (Baltimore). 2020 Aug 21;99(34):e21846. doi: 10.1097/MD.0000000000021846. PMID: 32846833; PMCID: PMC7447331.

205: Khalifeh MR, Redett RJ. The management of patients on anticoagulants prior to cutaneous surgery: case report of a thromboembolic complication, review of the literature, and evidence-based recommendations. Plast Reconstr Surg. 2006 Oct;118(5):110e-117e. doi: 10.1097/01.prs.0000221114.01290.85. PMID: 17016167.

206: Gooch MR, Gin GE, Kenning TJ, German JW. Complications of cranioplasty following decompressive craniectomy: analysis of 62 cases. Neurosurg Focus. 2009 Jun;26(6):E9. doi: 10.3171/2009.3.FOCUS0962. PMID: 19485722.

207: Su TM, Cheng CH, Chen WF, Hsu SW. Spontaneous healing and complete disappearance of a ruptured posterior inferior cerebellar artery dissecting aneurysm. J Neurosurg Pediatr. 2014 May;13(5):503-6. doi: 10.3171/2014.1.PEDS13412. Epub 2014 Feb 28. PMID: 24580645.

208: Cooke R. Time and timing in surgery. Proc R Soc Med. 1965 Dec;58(12):999-1004. doi: 10.1177/003591576505801201. PMID: 5852694; PMCID: PMC1899042.

209: Hendrick EB, Hoffman HJ, Humphreys RP. Trauma of the central nervous system in children. Pediatr Clin North Am. 1975 May;22(2):415-24. doi: 10.1016/s0031-3955(16)33141-8. PMID: 1124224.

210: Tsukagoshi J, Miura S, Yamada A. Challenges in management of a giant left ventricular pseudoaneurysm complicated by intracerebral hemorrhage. J Card Surg. 2020 Jul;35(7):1636-1637. doi: 10.1111/jocs.14698. Epub 2020 Jun 19. PMID: 32557864.

211: Cai SS, Mossop C, Diaconu SC, Hersh DS, AlFadil S, Rasko YM, Christy MR, Grant MP, Nam AJ. The "Crumple Zone" hypothesis: Association of frontal sinus volume and cerebral injury after craniofacial trauma. J Craniomaxillofac Surg. 2017 Jul;45(7):1094-1098. doi: 10.1016/j.jcms.2017.04.005. Epub 2017 Apr 25. Erratum in: J Craniomaxillofac Surg. 2017 Nov;45(11):1907. doi: 10.1016/j.jcms.2017.08.014. PMID: 28551409.

212: Mu S, Li C, Yang X, Wang Y, Li Y, Jiang C, Wu Z. Reconstructive Endovascular Treatment of Spontaneous Symptomatic Large or Giant Vertebrobasilar Dissecting Aneurysms: Clinical and Angiographic Outcomes. Clin Neuroradiol. 2016 Sep;26(3):291-300. doi: 10.1007/s00062-014-0369-4. Epub 2014 Dec 25. PMID: 25540817.

213: Lylyk P, Cohen JE, Ceratto R, Ferrario A, Miranda C. Endovascular reconstruction of intracranial arteries by stent placement and combined techniques. J Neurosurg. 2002 Dec;97(6):1306-13. doi: 10.3171/jns.2002.97.6.1306. PMID: 12507128.

214: Suh SW, Modi HN, Yang J, Song HR, Jang KM. Posterior multilevel vertebral osteotomy for correction of severe and rigid neuromuscular scoliosis: a preliminary study. Spine (Phila Pa 1976). 2009 May 20;34(12):1315-20. doi: 10.1097/BRS.0b013e3181a028bc. PMID: 19455007.

215: Gaio M, Ferrajolo C, Zinzi A, Riccardi C, Di Filippo P, Carangelo L, Pieretti G, Rossi F, Nicoletti GF, Capuano A. Association of Direct Oral Anticoagulants (DOACs) and Warfarin With Haemorrhagic Risk by Applying Correspondence Analysis to Data From the Italian Pharmacovigilance Database – A Case Study. Front Pharmacol. 2021 Dec 7;12:790740. doi: 10.3389/fphar.2021.790740. PMID: 34950040; PMCID: PMC8691542.
