# Supplementary material for: Intracranial Hemorrhage After Reduction Malarplasty: A Narrative Review Focusing on Surgical Technique
Source: J Clin Med. 2026 Jul 17;15(14):5609. doi: 10.3390/jcm15145609 (PMC13413184; doi:10.3390/jcm15145609)
Supplement: Supplementary file 1 [file jcm-15-05609-s001.zip › Supplementary Material S2.pdf]

Fifty-one papers that underwent full-text evaluation after screening of duplicate articles, titles, and abstracts

1: Zhang J, Liu H, Liu Y, Liu S, He Z, Chen G, Luo E. A Systematic Review and Meta-Analysis of Complications among Various Reduction Malarplasty. Aesthetic Plast Surg. 2023 Jun;47(3):1018-1038. doi: 10.1007/s00266-022-03104-1. Epub 2022 Oct 19. Erratum in: Aesthetic Plast Surg. 2023 Dec;47(6):2909. doi: 10.1007/s00266-023-03454-4. PMID: 36261745.

2: Lee JS, Lee JW, Yang JD, Chung HY, Cho BC, Choi KY. A rare complication of reduction malarplasty. Aesthetic Plast Surg. 2015 Apr;39(2):240-2. doi: 10.1007/s00266-014-0441-0. Epub 2015 Jan 22. PMID: 25608911.

3: Myung Y, Kwon H, Lee SW, Baek RM. Postoperative Complications Associated With Reduction Malarplasty via Intraoral Approach: A Meta Analysis. Ann Plast Surg. 2017 Apr;78(4):371-378. doi: 10.1097/SAP.0000000000000913. PMID: 27801697.

4: Moon JH, Lee GH. Fatal Meningitis Following Reduction Malarplasty: A Previously Unreported Complication. J Craniofac Surg. 2026 Jun 1. doi: 10.1097/SCS.0000000000013016. Online ahead of print

5.1097/SCS.0000000000013016. Epub ahead of print. PMID: 42223980.10: Rodman R. Cosmetic bone contouring. Curr Opin Otolaryngol Head Neck Surg. 2017 Aug;25(4):337-340. doi: 10.1097/MOO.0000000000000370. PMID: 28504986.

6: Zhang J, Liu H, Liu Y, Liu S, He Z, Chen G, Luo E. Correction: A Systematic Review and Meta-Analysis of Complications Among Various Reduction Malarplasty. Aesthetic Plast Surg. 2023 Dec;47(6):2909. doi: 10.1007/s00266-023-03454-4. Erratum for: Aesthetic Plast Surg. 2023 Jun;47(3):1018-1038. doi: 10.1007/s00266-022-03104-1. PMID: 37353599.

7: Gao ZW, Wang WG, Zeng G, Lu H, Ma HH. A modified reduction malarplasty utilizing 2 oblique osteotomies for prominent zygomatic body and arch. J Craniofac Surg. 2013 May;24(3):812-7. doi:

10.1097/SCS.0b013e31828dcd88. PMID: 23714886.

8: Chung S, Park S. Lowering Lateral Canthoplasty and Orbital Rim Shaving: An Ignored but Necessary Procedure for Maximizing the Effect of Reduction Malarplasty in Asians. Aesthetic Plast Surg. 2019 Jun;43(3):686-694. doi: 10.1007/s00266-019-01342-4. Epub 2019 Mar 21. PMID: 30903250.

9: Hsiao CW, Hsiao KY, Shen YD, Zavala A, Hsiao YW. Easy and Safe Simultaneous Zygoma Reduction and Facelift by Single Incision Through Subcutaneous Approach. J Plast Reconstr Aesthet Surg. 2022 Sep;75(9):3513-3520. doi:10.1016/j.bjps.2022.05.002. Epub 2022 Jun 15. PMID: 35821011.

10: Gao W, Qiu Y, Zou Y, Qiao C, Chang L, Jin Y, Chen H, Lin X. Reduction Malarplasty With Subperiosteal Lift Via a Single Limited Temporal Incision. Ann Plast Surg. 2021 Mar 1;86(3S Suppl 2):S194-S198. doi: 10.1097/SAP.0000000000002644. PMID: 33651014.

11: Han MD, Kwon TG. Zygoma and Mandibular Angle Reduction: Contouring Surgery to Correct the Square Face in Asians. Oral Maxillofac Surg Clin North Am. 2023 Feb;35(1):83-96. doi: 10.1016/j.coms.2022.06.003. Epub 2022 Nov 3. PMID: 36336603.

12: Tan W, Niu F, Yu B, Gui L. Feasibility of absorbable plates and screws for fixation in reduction malarplasty with L-shaped osteotomy. J Craniofac Surg. 2011 Mar;22(2):546-50. doi: 10.1097/SCS.0b013e318208bb41. PMID: 21403525.

13: Mu X. Experience in East Asian facial recontouring: reduction malarplasty and mandibular reshaping. Arch Facial Plast Surg. 2010 Jul-Aug;12(4):222-9. doi: 10.1001/archfacial.2010.48. PMID: 20644225.

14: Choi BK, Seo JY, Seo HJ, Choi SJ, Lee JW, Kim MW, Nam SB, Bae YC. Analysis and Guidelines for Revisional Malarplasty; Most Common Facial Skeletal Contouring Surgery. J Craniofac Surg. 2022 Sep 1;33(6):1674-1678. doi: 10.1097/SCS.0000000000008403. Epub 2021 Dec 7. PMID: 34879016.

15: Dong G, Teng L, Lu J, Huang Y. Application of the Bracing System in Reduction Malarplasty in Asian Population. Aesthetic Plast Surg. 2020 Feb;44(1):114-121. doi: 10.1007/s00266-019-01532-0. Epub 2019 Nov 7. PMID: 31701202.

16: Zou C, Wang JQ, Liu JF, Niu F, Chen Y, Wang M, Gui L. Reduction Malarplasty With Face-Lift for Older Asians With Prominent Zygoma. Ann Plast Surg. 2016 Aug;77(2):141-4. doi: 10.1097/SAP.0000000000000586. PMID: 26207549.

17: Hwang CH, Lee MC. Reduction malarplasty using a zygomatic arch-lifting technique. J Plast Reconstr Aesthet Surg. 2016 Jun;69(6):809-818. doi: 10.1016/j.bjps.2016.03.004. Epub 2016 Mar 23. PMID: 27084574.

18: Yang HW, Hong JJ, Koo YT. Reduction Malarplasty that Uses Malar Setback Without Resection of Malar Body Strip. Aesthetic Plast Surg. 2017 Aug;41(4):910-918. doi: 10.1007/s00266-017-0879-y. Epub 2017 May 23. PMID: 28536928.

19: Zhang Y, Tang M, Jin R, Zhang Y, Zhang Y, Wei M, Qi Z. Comparison of three techniques of reduction malarplasty in zygomaticus and massateric biomechanical changes and relevant complications. Ann Plast Surg. 2014 Aug;73(2):131-6. doi: 10.1097/SAP.0b013e318273f81f. PMID: 23407255.

20: Gao B, Yuan Y, Li K, Li Z, Yu L. Facial Contour Rejuvenation by Reduction Malarplasty Combined With Second-Stage Fat Grafting. J Craniofac Surg. 2021 Jan-Feb 01;32(1):179-183. doi: 10.1097/SCS.0000000000007043. PMID: 33196618.

21: Cho J, Kwon JS, Lee UL. Occlusion-Fit Three-Dimensional-Printed Zygoma Repositioner. J Craniofac Surg. 2018 May;29(3):731-732. doi: 10.1097/SCS.0000000000004315. PMID: 29419598.

22: Baek RM, Kim J, Lee SW. Revision reduction malarplasty with coronal approach. J Plast Reconstr Aesthet Surg. 2010 Dec;63(12):2018-24. doi: 10.1016/j.bjps.2010.01.007. Epub 2010 Feb 13. PMID:

20153991.

23: Ge H, Wang Y, Gao H, Sun X, Wu Y, Li J. The accuracy of virtual surgical planning assisted management for L-shaped reduction malarplasty: A retrospective study. J Craniomaxillofac Surg. 2024 Mar;52(3):363-368. doi: 10.1016/j.jcms.2024.01.016. Epub 2024 Jan 19. PMID: 38278743.

24: Kim JJ, Lee EY, Seok H, Kang JY. An improved technique for zygoma reduction malarplasty. J Craniomaxillofac Surg. 2018 Apr;46(4):654-659. doi: 10.1016/j.jcms.2018.01.010. Epub 2018 Feb 2. PMID: 29519577.

25: Kim JW, Hwang W. Optimal Fixation Location in Intraoral Reduction Malarplasty Using an L-Shaped Osteotomy. J Craniofac Surg. 2019 Nov-Dec;30(8):2490-2492. doi: 10.1097/SCS.0000000000005860. PMID: 31469740.

26: Kim T, Baek SH, Choi JY. Reduction malarplasty according to esthetic facial unit analysis: retrospective clinical study of 23 cases. J Oral Maxillofac Surg. 2014 Aug;72(8):1565-78. doi: 10.1016/j.joms.2014.02.029. Epub 2014 Feb 25. PMID: 24768421.

27: Li Q, Gao B, Li K, Xie F, Zhu H, Yu LG. A Novel Technique for Reduction Malarplasty by Inward Displacement of Infractured Zygomatic Arch Without Fixation. J Oral Maxillofac Surg. 2017 Dec;75(12):2658-2666. doi: 10.1016/j.joms.2017.06.022. Epub 2017 Jun 24. PMID: 28710911.

28: Lin LX, Yuan JL, Wang YT, Huang Y, Wang P, Wang XM. A New Infracture Technique for Reduction Malarplasty with an L-Shaped Osteotomy Line. Med Sci Monit. 2015 Jul 6;21:1949-54. doi: 10.12659/MSM.893503. PMID: 26145181; PMCID: PMC4501642.

29: Hwang K. Lateral rectus muscle injury, orbital fracture, mouth locking, and facial palsy resulting from reduction malarplasty. J Craniofac Surg. 2011 Jan;22(1):151-4. doi: 10.1097/SCS.0b013e3181f6fa0d. PMID: 21187757.

30: Qiao C, Xu J, Miao J, Shi Z, Yan K, Yan S, Qu Y, Wu G. Enhanced Midface Contouring: Simultaneous Zygomatic Reduction and Partial Coronoidectomy for Optimal Aesthetic and Functional Outcomes. Aesthetic Plast Surg. 2025 Oct;49(20):5725-5733. doi: 10.1007/s00266-025-05158-3. Epub 2025 Aug 13. PMID: 40801932.

31: Lee JS, Kim EH, Lee SH. Endoscopically assisted malarplasty: L-rotation technique. J Stomatol Oral Maxillofac Surg. 2021 Jun;122(3):229-234. doi: 10.1016/j.jormas.2020.07.011. Epub 2020 Aug 15. PMID: 32810601.

32: Al-Watary MQ, Hao J, He Y, Song L, Gao H, Alkebsi K, Elayah SA, Ye B, Li J. Evaluation of Different Fixation Methods Combinations After L-Shaped Osteotomy Reduction Malarplasty: An In Vitro Biomechanical Study. Aesthetic Plast Surg. 2024 Aug;48(16):3170-3179. doi: 10.1007/s00266-024-03960-z. Epub 2024 Mar 22. PMID: 38519573.

33: Kotha VS, Kanuri A, Mandelbaum M, Lakhiani C, Hung RW, Wang J, Rashid W, Chao JW. Simultaneous Zygomatic Osteotomies With Reduction Mandibuloplasty – An Approach to Mid- and Lower-Facial Feminization in the Transfeminine Patient. J Craniofac Surg. 2022 Jul-Aug 01;33(5):1569-1573. doi: 10.1097/SCS.0000000000008386. Epub 2021 Dec 1. PMID: 34855635.

34: Zhang Q, Chang C, Meng Z, Huang J, Guo J, Ge Z. Novel treatment of revision malarplasty with piezosurgery: A case report. Medicine (Baltimore). 2020 Oct 9;99(41):e22529. doi: 10.1097/MD.0000000000022529. PMID: 33031295; PMCID: PMC7544381.

35: Yang X, Mu X, Yu Z, Gu Q, Cao D, Yu D, Wei M, Chang T. Compared study of Asian reduction malarplasty: wedge-section osteotomy versus conventional procedures. J Craniofac Surg. 2009 Sep;20 Suppl 2:1856-61. doi: 10.1097/SCS.0b013e3181b6c65f. PMID: 19816365.

36: Baek RM, Kim J, Kim BK. Three-dimensional assessment of zygomatic malunion using computed tomography in patients with cheek ptosis caused by reduction malarplasty. J Plast Reconstr Aesthet Surg. 2012 Apr;65(4):448-55. doi: 10.1016/j.bjps.2011.10.019. Epub 2011 Nov 17. PMID: 22099146.

37: Kim TG, Cho YK. A New Double Trapezoid-Shaped Osteotomy for Reduction Malarplasty. J Craniofac Surg. 2016 Jan;27(1):87-93. doi: 10.1097/SCS.0000000000002268. PMID: 26703047.

38: Choi BK, Lee KT, Oh KS, Yang EJ. Preservation of the deep facial vein in reduction malarplasty. J Craniofac Surg. 2012 May;23(3):e254-7. doi: 10.1097/SCS.0b013e3182518845. PMID: 22627450.

39: Choung JW. Rotation technique of reduction malarplasty. J Craniofac Surg. 2015 Jan;26(1):238-9. doi: 10.1097/SCS.0000000000001199. PMID: 25569399; PMCID: PMC4297216.

40: Yuan J, Cho MY, Zhang Y, Qi ZL, Wei M. Influence of the maxillary sinus exposure in reduction malarplasty with an L-shaped osteotomy. J Craniofac Surg. 2011 Sep;22(5):1788-90. doi: 10.1097/SCS.0b013e31822e776b. PMID: 21959433.

41: Hwang CH, Lee MC. Revision malarplasty guided by strategic categorization. J Plast Reconstr Aesthet Surg. 2019 Feb;72(2):322-334. doi: 10.1016/j.bjps.2018.10.024. Epub 2018 Nov 13. PMID: 30514620.

42: Kim YH, Seul JH. Reduction malarplasty through an intraoral incision: a new method. Plast Reconstr Surg. 2000 Dec;106(7):1514-9. doi: 10.1097/00006534-200012000-00011. PMID: 11129179.

43: Wang T, Gui L, Tang X, Liu J, Yu D, Peng Z, Song B, Song T, Niu F, Yu B. Reduction malarplasty with a new L-shaped osteotomy through an intraoral approach: retrospective study of 418 cases. Plast Reconstr Surg. 2009 Oct;124(4):1245-1253. doi: 10.1097/PRS.0b013e31819e6562. PMID: 19935309.

44: Mahatumarat C, Rojvachiranonda N. Reduction malarplasty without external incision: a simple technique. Aesthetic Plast Surg. 2003 May-Jun;27(3):167-71. doi: 10.1007/s00266-003-0083-0. Epub 2003 Aug 21. PMID: 12925859.

45: Zhou J, Qi Z, Jin X. Simultaneous Surgery for Contouring the Prominent Zygoma and Mandibular Angles With Facelift in Middle-Aged Patients. J Craniofac Surg. 2020 Mar/Apr;31(2):448-452. doi: 10.1097/SCS.0000000000006227. PMID: 31977701.

46: Younis H, Zhou Z, Sun X, Ge H, Wang Y, Li J. Middle and lower-facial feminization surgery in East Asian transgender and cisgender women: Surgical techniques and outcomes. J Plast Reconstr Aesthet Surg. 2025 May;104:215-224. doi: 10.1016/j.bjps.2025.03.024. Epub 2025 Mar 12. PMID: 40154114.

47: Hong SE, Liu SY, Kim JT, Lee JH. Intraoral zygoma reduction using L-shaped osteotomy. J Craniofac Surg. 2014 May;25(3):758-61. doi: 10.1097/SCS.0000000000000759. PMID: 24657982; PMCID: PMC4025629.

48: He Y, Wang Y, Al-Watary MQ, Wang Y, Wu Y, Li X, Ye B, Li J. The L-Shaped Zygomatic Reduction with Oblique or Vertical Resection: Which One Is the Optimal Choice? Plast Reconstr Surg. 2025 Jan 1;155(1):26e-34e. doi: 10.1097/PRS.0000000000011396. Epub 2024 Mar 12. PMID: 38470996.

49: Liao L, Hsu Y, Hu J, Li X, Li H, Li J. Correction of asymmetric facial deformity by contouring: indications and outcomes. J Craniofac Surg. 2015 Mar;26(2):e94-8. doi: 10.1097/SCS.0000000000001026. PMID: 25643344.

50: Zou C, Niu F, Liu JF, Yu B, Chen Y, Wang M, Gui L. Application of Computer Techniques in Correcting Mild Zygomatic Assymetry With Unilateral Reduction Malarplasty. J Craniofac Surg. 2015 Sep;26(6):2002-4. doi: 10.1097/SCS.0000000000001894. PMID: 26359703.

51: Shao Z, Xie Y, Yu B, Liu L, Du T. A new assisted fixation technique to prevent zygoma displacement in malar reduction. Aesthetic Plast Surg. 2013 Aug;37(4):692-6. doi: 10.1007/s00266-012-0033-9. Epub 2013 Jan 8. PMID: 23296763.
